# The impact of haplotypes derived from Chinese pigs on genetic variation and economic traits in the Duroc breed

**DOI:** 10.1186/s12711-025-01010-z

**Published:** 2025-10-23

**Authors:** Suyun Fang, Chao Guo, Hang Liu, Yuzhan Wang, Cheng Tan, Zhenfang Wu, Yiqiang Zhao, Xiaoxiang Hu, Ruifei Yang

**Affiliations:** 1 College of Animal Science and Technology, Yunnan Agricultural University, Kunming, 650201 Yunnan China; 2 State Key Laboratory of Agrobiotechnology, College of Biological Sciences, China Agricultural University, Beijing, 100193 China; 3 Key Laboratory of Genetic Evolution & Animal Models and Yunnan Key Laboratory of Molecular Biology of Domestic Animals, Kunming Institute of Zoology, Chinese Academy of Sciences, Kunming, 650223 Yunnan China; 4 School of Life Sciences, Division of Life Sciences and Medicine, University of Science and Technology of China, Hefei, 230026 Anhui China; 5 Kunming College of Life Science, University of Chinese Academy of Sciences, Kunming, 650204 Yunnan China; 6 National Engineering Research Center for Breeding Swine Industry, South China Agricultural University, No. 483 Wushan road, Tianhe district, 510640 Guangdong China

## Abstract

**Background:**

The historical importation of Chinese pigs into Western countries has facilitated the introduction of Chinese haplotypes into European pig breeds, thereby shaping their genetic diversity and phenotypic traits. However, the genetic and biological implications of this introgression remain poorly understood.

**Results:**

Based on SNP chip and resequencing data, we confirmed significant genetic introgression from Chinese pigs into commercial European lines. The genetic origins of the introgressed segments predominantly derive from Southern Chinese domestic pigs (CSDP), with additional contributions from other populations, such as Eastern Chinese domestic pigs (CEDP). Our study demonstrates that the selection pressure for Chinese pig introgression was stronger in Duroc pigs compared to the Large White and Landrace breeds. Based on ancestral haplotypes from CEDP and CSDP, we conducted a genome-wide association study (GWAS) and identified 10 quantitative trait loci (QTLs), five of which were not identified in previous studies or using SNPs. Expression genome-wide association studies (eGWAS) based on these introgressed haplotypes, using gene expression profiles from the duodenum, liver, and muscle tissues in the Duroc population, revealed eGWAS signals that were enriched near transcript start sites. By integrating GWAS signals for loin muscle depth with eGWAS signals in muscle tissue, we confirmed that a region 300 Kb from *TAF11*, which is enriched with open chromatin regions and encompasses a super-enhancer located within the same topologically associating domain as *TAF11*, was associated with both *TAF11* expression and loin muscle depth, highlighting the profound influence of Chinese introgression.

**Conclusions:**

These findings offer valuable insights into the genetic influences of Chinese pig introgression on the Duroc breed, as well as the molecular basis for its effects on economically important traits in Duroc pigs.

**Supplementary Information:**

The online version contains supplementary material available at 10.1186/s12711-025-01010-z.

## Background

Pigs are among the most significant farm animals, providing a wide array of meat products worldwide [1]. Since their domestication approximately 10,000 years ago, pigs have undergone extensive local adaptation and artificial selection, resulting in the differentiation into European and Asian breeds [2]. During the 18th and 19th centuries, Western countries imported Chinese pigs and hybridized them with local European breeds [3]. These efforts, facilitated by intensive selective breeding, culminated in the development of modern commercial pig breeds [4, 5]. Over centuries of selection, the production and growth traits of commercial lines have proven to be far superior to those of local breeds. Notably, introgressed genes from Chinese pigs appear to have been selectively favored. For instance, the Asian-derived haplotype associated with litter size at the *AHR* locus showed evidence of selection in Large White pigs, as reproductive traits were a primary breeding goal in 19th-century European countries [3, 4]. Other introgressions from Chinese pigs into European breeds have also been identified, such as *LEMD3* related to ear morphology and *VRTN* associated with vertebra number, resulting in modern commercial lines that possess Chinese ancestral alleles compared to European indigenous pigs [6, 7].

The Duroc pig is one of the most widely used breeds in the commercial pig industry and originated in the United States in the late nineteenth century. This breed is renowned for its high growth rate and high-quality carcasses with great marbling, making significant contributions to modern pork production [1]. In comparison to other commercial lines, such as Large White and Landrace, the Duroc breed has experienced a more intense selection process, as it is commonly used as the terminal sire in three-way hybrid breeding systems.

The historical events of introduction of Asian pigs into Europe has been well documented [4], suggesting that the Chinese introgression is likely widespread across European breeds. Moreover, certain introgressed segments from Chinese pigs may have undergone stronger selection in Duroc pigs compared to other European commercial lines (ECP). Although some gene flow events have been identified in the Duroc breed [8], a comparative study is still lacking to investigate Chinese ancestry-derived genetic components in different ECP lines, and it remains unclear what these components are and how they affect economic trait performance.

In this study, we utilized previously published SNP chip and whole genome resequencing data [9–11] to trace the origin of genetic introgression from Chinese pigs into Duroc populations. We conducted a comparative analysis of introgression with other commercial lines to further explore the selection pressure on the Chinese haplotypes. Furthermore, based on low-coverage genome sequencing data and RNA-seq data derived from the Duroc breed, we performed a genome-wide association study (GWAS) and an expression genome-wide association study (eGWAS) to investigate the associations of the Chinese haplotype with phenotypes and their impact on gene expression profiles. Finally, we combined Hi-C, ATAC-seq, and ChIP-seq signals with co-localization results to conduct an integrative analysis aimed at accurately exploring how the introgressed loci influence muscle traits. Collectively, we aim to conduct a comprehensive exploration of the introgression from Chinese pigs into the Duroc breed, highlighting their contributions to phenotypic performance and expression profiles, while demonstrating the molecular basis of their significant impact on economic traits.

## Methods

### Data collection

We collected various datasets for conducting genetic introgression analyses, including SNP chip data (https://datadryad.org/stash/dataset/doi:10.5061/dryad.30tk6) [9], which was processed (genomic coordinates transferred to the coordinates of Sscrofa11.1) and provided by Wang et al. [10], and genome resequencing data of 937 pigs worldwide (https://ngdc.cncb.ac.cn/gvm/getProjectDetail?%20project=GVM000479) [11]. Both data sets were obtained as VCF files. We used the low-coverage genome resequencing data of Duroc pigs to perform GWAS analyses, using genotype and phenotype data from the same population. The genotype data (https://gigadb.org/dataset/view/id/100894/) have been described and published by [12]. The corresponding phenotype data were reported by [12–14], encompassing backfat thickness (BF), teat number (TN), loin muscle depth (LMD), and loin muscle area (LMA). The phenotype records for litter size (LS) were sourced from female parents from the same litter. We also utilized genome resequencing data and corresponding transcriptomic sequencing data of Duroc pigs from muscle, liver, and duodenum tissues (NCBI accession: PRJEB58030) [15]. We also included ATAC-seq and ChIP-seq data from muscle tissue of Meishan, Duroc, Large White, and Enshi pigs, along with Hi-C data from a Large White pig (see Additional file 1: Table S1) [16]. The processed ATAC-seq data, including bigwig files representing signals and bed files representing narrow peaks, were downloaded from the GEO database (GSE143288).

### Genomic data pre-processing

Conversion of coordinates for the SNP chip data was conducted in accordance with the methodology described in [10]. Subsequently, we utilized VCFtools (v0.1.17) to filter SNPs using the following parameters: --maf 0.05 --max-alleles 2 --min-alleles 2 --max-missing 0.95 [17]. We also excluded sites located on sex chromosomes, mitochondrial DNA, and uncertain chromosomes. We included Chinese indigenous pigs, ECPs, European domestic pigs (EDP, including Manglica and Iberian breeds), European wild boars (EWB), and an outgroup made up of related species in this study, based on the results of population clustering, admixture results, and the phylogenic tree of these pig breeds reported in previous studies [9, 10], and according to the historical records in relation to latitude, longitude, and distribution of these pigs [1], as well as our research purpose of analyzing Chinese introgression into ECP lines. The Chinese pig samples consisted of 70 Northern Chinese domestic pigs (CNDP), 143 Southern Chinese domestic pigs (CSDP), 95 Mid-Chinese domestic pigs (CMDP), 71 Eastern Chinese domestic pigs (CEDP), and 70 Southern Western Chinese domestic pigs (CSWDP). The European sample included 285 ECPs, 177 EWBs, and 40 EDPs. The outgroup consisted of 39 individuals from related species (Babyrousa babyrussa, Phacochoerus africanus, Sus barbatus, Sus celebensis, and Sus verrucosus) (see Additional file 1: Table S2). In total, 990 individuals with 50,705 SNPs were retained for further analysis.

The high depth resequencing dataset (HD dataset), was obtained by downloading the VCF files for 937 pigs from around the world, as well as from resequencing data for 14 warthogs (see Additional file 1: Table S3). For the warthog data, we utilized the GTX platform from Genetalks, which is a Field Programmable Gate Array (FPGA)-based hardware accelerator [18], to perform genome alignment and variant calling using the Sscrofa11.1 reference genome, informed by the SNP sites from the resequencing data of the 937 pigs. We filtered out sites with missing alleles in more than one individual and employed Beagle software (v5.4) for phasing and imputation [19]. Subsequently, we merged the VCF files using bcftools (v1.21) and retained individuals for downstream analysis [20], which included 102 CSDPs, 93 CNDPs, 138 CEDPs, 43 Chinese wild boars (CWB), 121 ECPs, 53 crossbred lines (specifically the three-line crossbred EDLY and White Duroc: EWDU), and 14 EWB, while filtering out variants with a minor allele frequency (MAF) less than 0.01. This process resulted in a final dataset of 578 pigs with 16,549,697 autosomal variants available for analysis (see Additional file 1: Table S3). We also downloaded resequencing data from 100 Duroc pigs for the eGWAS [15]. The genome alignment and variant calling procedures for these pigs were consistent with the aforementioned methods, and the quality control was conducted using VCFtools (v0.1.17) with the following parameters: --maf 0.05, --min-alleles 2, --max-alleles 2, --remove-indels, --hwe 1e-4, and --max-missing 0.95 [17]. For phasing and imputation of the Duroc pigs, we again used Beagle software (v5.4) based on the reference panel derived from the resequencing data of the 937 pigs [19], resulting in a final count of 7,437,797 SNPs.

For the low coverage genome resequencing data (LCS dataset), we retained CEDP, CSDP, and EWB pigs from a total of 937 pigs and combined these with downloaded LCS data from 2802 Duroc pigs [12]. Subsequently, we filtered variants with a MAF of less than 0.01 and only retained SNP sites detected in both the LCS data and in the 937 pigs. Beagle software (v5.4) was then used for imputation and phasing based on the reference panel derived from the resequencing data of the 937 pigs, resulting in a final dataset of 3,056 pigs with 7,436,569 SNPs.

### Transcriptomic data pre-processing

After downloading the raw data from the NCBI database, Trimmomatic (v0.39) was employed for data quality control [21]. The filtered paired-end reads were then mapped to the transcriptome using Bowtie2 (2.3.4.3) [22]. Reads that mapped to the forward strand (parameters: -F 4 -F 16) and the reverse strand (parameters: -F 4 -f 16) were counted using SAMtools (v1.5) [23]. Subsequently, the direction of chain specificity was determined based on the proportion of reads mapped to sense strand chains. A proportion greater than 0.8 in read1 (i.e., the first read of the paired-end fragment in FASTQ format) and less than 0.2 in read2 (the second read of the pair) were classified as stranded, whereas a proportion less than 0.2 in read1 and greater than 0.8 in read2 were classified as reverse-stranded. The filtered reads were then compared to the genome using HISAT2 (v2.2.1) [24], with chain-specific parameters that were derived from the previous step. These samples contained multiple SRAs (Sequence Read Archives), which were retained if all SRAs in a sample exhibited a proportion of reads mapped to strand chains greater than 0.5 in read1 and less than 0.5 in read2. The gene expression matrix was quantified using StringTie (v2.1.2) [25], employing the transcripts per million (TPM) value for quantification. In the final step, we filtered genes if the proportion of samples with TPM values less than 0.1 exceeded 20% of all samples. As a result, we retained matrices consisting of 97 muscle samples with 12,143 TPMs, 97 duodenum samples with 14,586 TPMs, and 92 liver samples with 13,065 TPMs for subsequent downstream analysis.

### ChIP-seq data processing and super-enhancer identification

Raw ChIP-seq data from the muscle of 2-month-old Duroc, Large White, Meishan, and Enshi pigs were downloaded from the SRA database (see Additional file 1: Table S1) [26]. The transformed ChIP-seq FASTQ sequences were processed using the locally deployed ENCODE ChIP-seq pipeline (v.2.2.2) (https://github.com/ENCODE-DCC/chip-seq-pipeline2). Data pre-processing steps included trimming reads, aligning them to the reference pig genome (Sscrofa11.1) using BWA mem (v.0.7.17) [27], filtering out reads with low mapping quality using SAMTools (v.1.9) [23], and removing duplicates with Picard (v.2.20.7) (https://broadinstitute.github.io/picard). The filtered H3K27ac BAM files were utilized to identify super-enhancers. The ‘super’ module in Homer (v.4.11) was employed to detect super-enhancers marked by H3K27ac [28], with the maximum distance between flanking regions of two single pre-stitched peaks set at 12.5 Kb. H3K27ac peak clusters with signal slopes greater than one were classified as super-enhancer regions [29].

### Hi-C data processing

Hi-C data from muscle tissue of a Large White pig were obtained from the SRA database (see Additional file 1: Table S1) [16]. Following trimming with Fastp software [30], the Hi-C raw sequencing data were aligned to the pig reference genome (Sscrofa11.1) using BWA mem with the parameters -SP -A1 -B4 -E50 -L0, while all other parameters were set to default [27]. The aligned SAM files were converted to pair files using the pairtools (v1.0.2) ‘parse2’ tool [31], with additional settings: --drop-sam --drop-seq --add-columns mapq –flip. Sorting was conducted using the ‘sort’ function, and duplicates were removed with the ‘dedup’ tool. The filtered pairs were then loaded into a cool matrix using the cooler (v.0.9.1) ‘cload pairs’ tool [32]. The pairs were also loaded into a contact matrix utilizing the ‘pre’ module in juicertools (v.1.22.01) [33], while the ‘hiccups’ module was employed for loop calling.

### Global ancestry and local ancestry inference

To confirm the genome-wide admixture between ECP and Chinese indigenous pigs, we used the *D*-statistic to detect global genetic introgression signals. The *D*-statistic is calculated based on the counts of site patterns in a four-taxon phylogeny as follows:

1 $$D = \frac{{n_{{ABBA}} - n_{{BABA}} }}{{n_{{ABBA}} + n_{{BABA}} }}$$

where ${n}_{ABBA}$ and ${n}_{BABA}$ are the number of biallelic sites exhibiting ABBA and BABA patterns, respectively; ABBA sites represent allele sharing between the putative donor population (P3) and the test population (P2), indicating potential introgression from P3 into P2, while BABA sites reflect allele sharing between P3 and the reference population (P1), serving as a baseline for ancestral allele sharing. Based on the SNP chip data and high-depth resequencing data described above, we performed the *D*-statistic test using the Dsuite software (v0.5 r52) [34]. In this analysis, we designated Duroc, Large White, and Landrace commercial European pigs as the P2 population, while closely related European wild boars or domestic pigs (European Hungary Manglica pig: EHUMA or European Spanish Iberian pig: ESIB) served as the P1 population (sister group) for the SNP chip data, and EWB served as P1 population for the genome resequencing data. A Chinese pig population (CEDP, CSDP, CNDP, CMDP, or CSWDP) was designated as P3, while the previously described outgroup population was used for SNP chip data, with warthogs serving as the outgroup for genome resequencing data. Based on the Eq. (1), we performed *D*-statistic tests under the phylogeny ((P1, P2), P3, O) to detect excess allele sharing between P2 and P3 relative to P1. A positive *D*-statistic with a Z-score > 2 and a *p*-value < 0.05 was considered evidence of significant gene flow between the P2 and P3 groups. To validate the *D*-statistic results and to further illustrate allele sharing between Chinese pigs and the EDP, ECP, and EWB groups from SNP chip data, we calculated the Weir and Cockerham F_ST_ values of variants within 50 Kb windows (step size: 25 Kb). These values were used to compare the genetic distance between Chinese (CEDP and CSDP) and European groups (EDP, ECP and EWB) based on F_ST_, which was computed using VCFtools (v0.1.17). To assess whether the genetic distances significantly differed among group comparisons, we performed Duncan’s multiple range test based on the average F_ST_ values between each Chinese-European pair. Inference on admixture time between ECP and the Chinese groups (CEDP, CMDP, CSWDP, CSDP) was conducted using the ALDER tool (v1.03) [35], with one generation corresponding to five years.

To confirm the global ancestry signals detected previously, we utilized the RFMix (v2.03) software to quantify the introgressive hybridization of genomic segments from CEDP and CSDP into ECP (Duroc, Large White, or Landrace) [36], based on the resequencing data. For this analysis, the target population consisted of genetic material from the reference population, where the EWB, CEDP, and CSDP groups comprised the reference population in both the HD and LCS dataset analyses, while ECP and crossbred lines (EDLY and EWDU) were designated as the target population in the HD dataset. Duroc was considered as the target population in the LCS dataset. Considering non-overlapping haplotype blocks of 5 SNPs, haplotype block was classified to originate from a specific population if its probability for that population exceeded 0.5. Specifically, each haplotype was encoded as ‘0’ if it was of European pig origin, or as ‘1’ if it originated from either CEDP or CSDP. All subsequent analyses were performed using both the SNP data and this recoded haplotype dataset.

### Population selective sweep analysis and comparison of genetic introgression

To investigate the selective sweep intensity within genetic introgressed regions, EWB was selected as the comparative population in the pairwise analyses. Consequently, based on the HD dataset comprising 578 pigs, three pairwise analyses (Duroc vs. EWB; Large White vs. EWB; Landrace vs. EWB) were employed. The indicators of selective sweep signals utilized in this study were XP-EHH and iHS. Scores for XP-EHH and iHS were obtained using Selscan (v1.2.0a) and normalized across 50 Kb window bins throughout the entire genome [36]. For each bin, the percentages of variants with XP-EHH > 2 and |iHS| > 2 were recorded, while those with a minimum XP-EHH score > 2 within a bin and a top 1% normalized iHS score were classified as regions under positive selection. To further validate the positive selection signals, the Weir and Cockerham weighted F_ST_ values of variants within 50 Kb windows were analyzed, with F_ST_ values computed using VCFtools (v0.1.17) [17].

Based on the recoded dataset of haplotype blocks described above, we used VCFtools (v0.1.17) to calculate the frequency of the haplotypes from Chinese groups (CEDP and CSDP) [17] and the percentages of CEDP or CSDP introgression were computed as the average frequency of the haplotype from the CEDP or CSDP group in the ECP and crossbred lines. Regions with haplotype frequencies exceeding 0.5 were considered as introgressed regions from CEDP or CSDP. We compared the XP-EHH, iHS and F_ST_ indicators in these regions against genome-wide levels. The Chi-square test was used to show whether there were significant differences in the enrichment of positive selective bins within the introgressed bins derived from the CEDP and CSDP populations among Duroc, Landrace, and Large White groups. To investigate the correlation of Chinese introgression haplotype frequencies in ECP (Duroc, Large White, and Landrace) and European crossbreed lines (EDLY and EWDU), we fitted the data from ECPs with crossbreed lines (DLY and WDU) using the “glm” smooth method implemented in the “ggplot” R package (v3.5.2) (https://ggplot2.tidyverse.org/). Additionally, Pearson correlation coefficients and Kullback–Leibler divergence (KLD) were calculated to assess the similarity in haplotype frequencies derived from the CEDP and CSDP populations between the ECP and the crossbred lines. Both analyses were performed using R software (v4.2.2).

### Local ancestry haplotype-based genome-wide association study

As illustrated in previous studies [37, 38], GWAS analysis integrate with ancestral haplotype information has the advantage of combining linked SNPs to control false positives and capture short-range interactions, but also enhances statistical power by grouping haplotypes into few clusters based on ancestral origins. This study was based on the LCS dataset, with ancestral haplotype information incorporated using the corresponding recoded data described above. We designed statistical models for various types of traits, with all computations across the whole genome conducted using the GCTA package [39]. For the traits BF, LMA, and LMD, birth year, month, and parity were included as discrete covariates, while weights at the beginning and end of the test served as quantitative covariates. In contrast, for the traits TN and LS, only year, month, and parity were included as discrete covariates using the statistical model:

2 $$\mathbf{y}=\mathbf{X}\varvec{\upbeta}+\varvec{\upalpha}\mathbf{b}+\mathbf{W}\mathbf{g}+\mathbf{e}$$

where $\mathbf{y}$ is the vector of phenotypic observations; $\varvec{\upbeta}$ is the vector of fixed effects, or fixed effects and quantitative covariates, as described above; $\varvec{\upalpha}$ is the effect of a candidate haplotype and $\mathbf{b}$ the corresponding vector of recode haplotypes, with the effect of each haplotype evaluated separately; $\mathbf{g}$ is the vector of random polygenic additive effects, i.e. the cumulative effect of all SNPs, as captured by the genomic relationship matrix (GRM); $\mathbf{e}$ is the vector of random residuals; and $\mathbf{X}$ and $\mathbf{W}$ are the design matrices connecting phenotypes to covariables and random polygenic effects, respectively. The GRM was calculated using the recoded haplotypes, as implemented in the GCTA software [39]. To correct for multiple testing across the genome, the false discovery rate (*FDR*) correction was applied using the FDRtool R package (v1.2.18) to establish the genome-wide significance threshold (*FDR* < 0.05) [40]. The candidate quantitative trait locus (QTL) interval was defined based on the boundaries of ancestral introgressed haplotypes that exceeded the genome-wide significance threshold. Since the LS trait in this population was not investigated in previous studies [12, 13], we also conducted a SNP-based GWAS using the PLINK binary files and GRM matrices from a linkage disequilibrium–pruned dataset that was previously used in GWAS [12], which was downloaded from GigaDB (https://gigadb.org/dataset/view/id/100894). This analysis was performed using the same statistical model as described above.

Using the downloaded data, we also employed a genomic best linear unbiased prediction (GBLUP) model to calculate the genomic estimated breeding value (GEBV) for each individual using GVCBLUP software [41] based on the following model:

3 $$\mathbf{y}=\mathbf{X}\mathbf{b}+\mathbf{Z}\mathbf{g}+\mathbf{e}$$

where $\mathbf{y}$ is the vector of phenotypic observations; $\mathbf{b}$ is the vector of fixed effects, or fixed effects and quantitative covariates, including year, season, parity, and weights at the beginning and end of the test effects; $\mathbf{X}$ is the incidence matrix for $\mathbf{b}$; $\mathbf{g}$ is the vector of random polygenic additive effects, i.e. the accumulated effect of all SNPs, as captured by the genomic relationship matrix (GRM); $\mathbf{Z}$ is an incidence matrix allocating polygenic effects to phenotypic observations; and $\mathbf{e}$ is the vector of random residuals. The GEBV calculation was conducted. To validate the identified genetic differences in significant bins between Duroc pigs with and without ancestry haplotypes derived from Chinese pigs, we selected the top 100 pigs with the highest GEBVs (high group) and the lowest GEBVs (low group).

To further investigate whether the genetic differences in the introgressed bins from Chinese pig populations correspond to population-level divergence at the sequence level, we conducted a locus-specific branch length (LSBL) analysis for each SNP located within the candidate QTL regions identified from the ancestral haplotype-based GWAS, and calculated the LSBL values based on [42] as:

4 $$\varvec{LSBL}=\frac{({\varvec{d}}_{\varvec{high}-\varvec{low}}+{\varvec{d}}_{\varvec{high}-\varvec{CDP}}-{\varvec{d}}_{\varvec{low}-\varvec{CDP}})}{2}$$

where ${\varvec{d}}_{\varvec{high}-\varvec{low}}$, $\:{\varvec{d}}_{\varvec{high}-\varvec{CDP}}$, and $\:{\varvec{d}}_{\varvec{low}-\varvec{CDP}}$ are the pairwise F_ST_ values between the high and low group, the high group and Chinese domestic pigs (CDP: representing for CSDP or CEDP), and the low group and CDP groups, respectively. We identified 10 SNPs with the highest LSBL values in each of the candidate QTL regions and used them as representative sites for haplotype construction. Subsequently, we calculated the frequencies of these haplotypes in each population. A haplotype network based on the 10-SNP haplotypes was constructed using the minimum spanning network method implemented in PopART software (v1.7) [43].

### Expression genome-wide association study

Based on the recorded haplotype data of 100 Duroc pigs from the HD dataset, along with their corresponding gene expression matrices from muscle, liver, and duodenum tissues [15], we conducted a local ancestry haplotype-based eGWAS across thegenome. The statistical model for the eGWAS was similar to that of the GWAS model:

5 $$\mathbf{y}=\mathbf{X}\varvec{\upbeta}+\varvec{\upalpha}\mathbf{b}+\mathbf{W}\mathbf{g}+\mathbf{e}$$

where $\mathbf{y}$ is the vector of TPMs; $\varvec{\upbeta}$ is the vector of month age effect, which was the fixed factor; $\varvec{\upalpha}$ is the effect of a candidate haplotype or SNP, and $\mathbf{b}$ is the corresponding vector of recode haplotypes; $\mathbf{g}$ is the vector of random polygenic additive effects, i.e. the accumulated effect of all haplotypes; $\mathbf{e}$ is the vector of random residuals; and $\mathbf{X}$ and $\mathbf{W}$ are the design matrices connecting TPMs to covariables and random polygenic effects respectively. All eGWAS calculations were conducted using FastQTL (v2.184) [44], with permutations for multiple testing set to 1000 (maximized number set to 10,000) and the window size configured to 2 Mb. In the final step, *p*-values were corrected using the Bonferroni method, where the significance level (alpha = 0.05) was divided by the number of tested genes in each tissue (muscle: 12,143; liver: 13,065; duodenum: 14,586). Associations with Bonferroni-adjusted *p*-values < 0.05 were considered statistically significant.

### Co-localization of the TAF11 locus

To further investigate the association of CEDP introgression with *TAF11* expression, and the loin muscle depth trait, we expanded the QTL region from 28 to 32 Mb on Chromosome 7. To investigate whether the same genetic variants within the introgressed regions are associated with both gene expression (eQTLs) and in the trait, as identified in GWAS (QTLs), we used the coloc R package (v5.2.3) and SMR software (v1.3.1) to conduct co-localization analysis [45, 46]. The coloc method uses a Bayesian framework to estimate the posterior probability that the same variant affects both traits (i.e., PP.H4), while the SMR method applies a summary-data-based Mendelian randomization model to test the association between gene expression and the complex trait based on shared instrumental variables. By integrating results from coloc and SMR, we aimed to cross-validate shared signals between eQTLs and GWAS hits, thereby increasing the robustness of our candidate variant identification. For the coloc analysis, we first retrieved the eGWAS results derived from FastQTL analysis, which spanned from 28 to 32 Mb on Chromosome 7. Subsequently, we used the LCS data and Plink (v1.90) to calculate the linkage disequilibrium (LD) (r²) between the most significant haplotype block associated with loin muscle depth (Chr7:30,389,320 − 30,392,246) and other blocks in this region [47]. For each coloc run, haplotype blocks with high LD (r² > 0.9) were added separately to the remaining non-redundant sites (one at a time) and tested individually using the coloc R package. After conducting all coloc tests, we combined the results and retained only the highest PP.H4 value for each site. PP.H4 represents the probability that the same causal variant underlies both the GWAS and eQTL signals. We considered sites with PP.H4 > 0.75 as significant haplotypes in the coloc analysis. For the SMR analysis, we similarly defined the region on Chromosome 7 and tested each site based on GWAS and eGWAS results, applying a significance threshold of *p*-value < 1e-5. The analysis for SNPs in both coloc and SMR followed the same protocol as described above. To validate the presence of Chinese introgression, we constructed a maximum-likelihood tree using Treemix (v1.13) [48], based on SNPs within the co-localized region, among the following groups: Duroc pigs with the 100 highest (DUROC-h) and 100 lowest GEBVs (DUROC-l) for LMD, EWB, and Chinese indigenous pigs (CEDP, CNDP, CSDP). A heatmap of haplotypes derived from these SNPs was also plotted to compare the differences among these populations. Subsequently, the co-localized results were further integrated with data from ATAC-seq, ChIP-seq, and Hi-C outputs for the same region. The data used for Hi-C interaction heatmaps and topologically associating domain (TAD) results presentation were derived from our processed cool files and TAD domain files. The final visualization of multiple omics data on genome browser tracks was integrated using pyGenomeTracks (v.3.8) [49].

## Results

### Global genetic introgression from Chinese pigs into European commercial lines

**Fig. 1:**
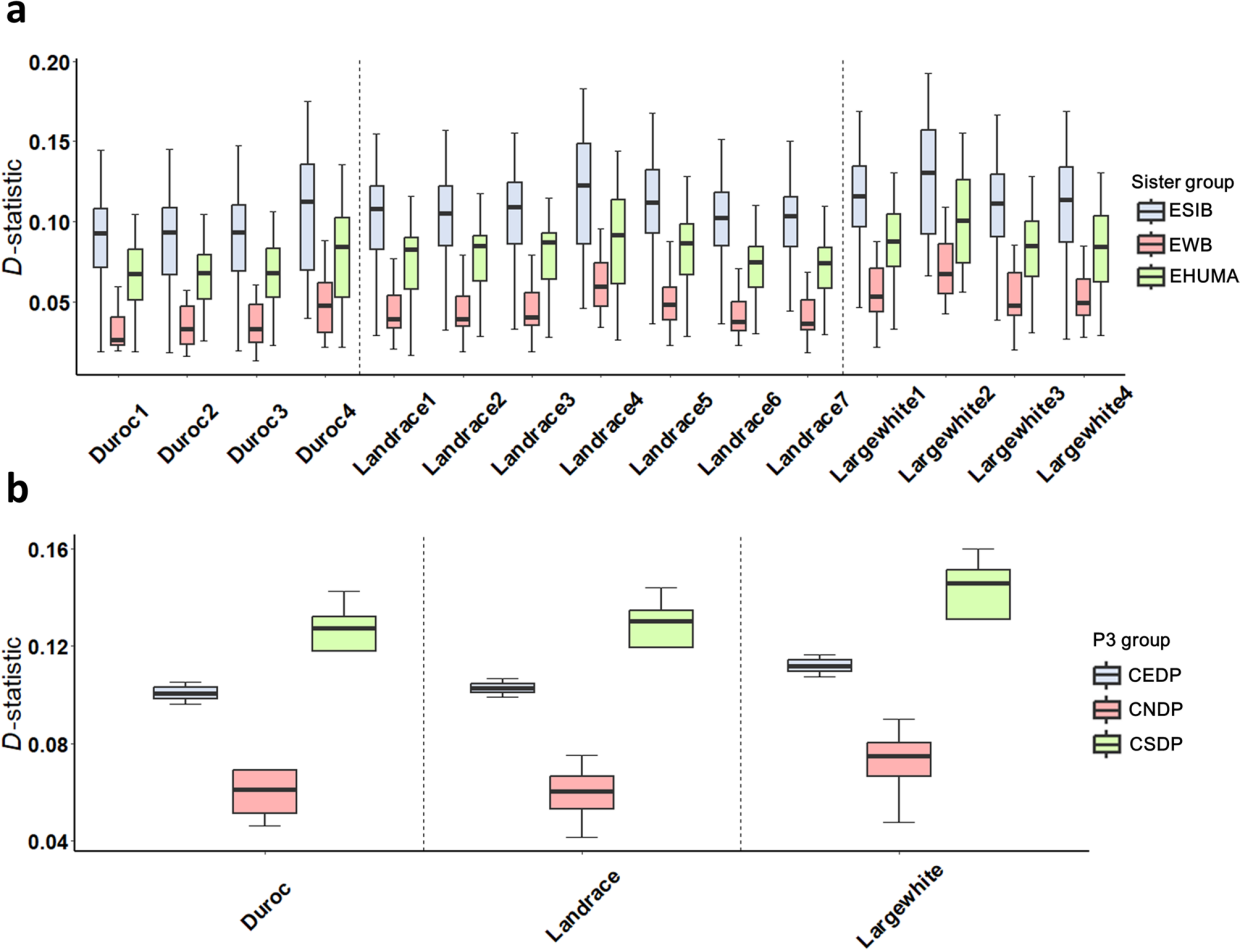
D-statistics with D derived based on a P1 group (European indigenous domestic pigs or European wild boars), a P2 group (European commercial lines), a P3 group (Chinese domestic pigs), and an Outgroup. **a** *D*-statistics based on SNP chip data, with the x-axis representing P2 groups, including the Duroc breed (Duroc) from four producing regions (Duroc1: Denmark; Duroc2: USA; Duroc3: Netherlands; Duroc4: China), the Landrace breed (Landrace) from seven producing regions (Landrace1: Denmark; Landrace2: Norway; Landrace3: Finland; Landrace4: China; Landrace5: USA; Landrace6: Spain; Landrace7 Netherlands), and the Large White breed (Large White) from four producing regions (Large White1: Denmark; Large White2: China; Large White3: USA; Large White4: Netherlands). European pigs—including ESIB (European Iberian pig), EWB (European wild boar), and EHUMA (European Hungarian Mangalica pig)—were designated as the P1 (sister) group, while Chinese breeds were used as the P3 group. The outgroup was composed of Babyrousa babyrussa Babyrousa babyrussa, Phacochoerus africanus, Sus barbatus, Sus celebensis, and Sus verrucosus. The horizontal line in the boxplot represents the median value, and the legend depicts the corresponding P1 sister group used in *D*-statistic analysis (ESIB: European Iberian pig; EWB; EHUMA: European Hungary Mangalica pig). **b** *D*-statistics based on resequencing data. The *D* values were calculated using different P3 groups (indicated in the legend), including CEDP, CSDP, and CNDP. EWB (European wild boar) was set as the P1 group, while Duroc, Landrace, and Large White pigs were used as the P2 group. *Phacochoerus africanus* served as the outgroup

To investigate the genetic evidence of introgression from Chinese pigs into ECP populations, we first performed *D*-statistic analyses based on SNP chip data, using genome-wide SNPs to compare ECP lines (Duroc, Large White and Landrace) from different global production regions (see Additional file 1: Table S4). Using ESIB, EHUMA, and EWB as P1 groups (sister group), significant Chinese introgression signals were identified in all three lines from the different production regions (Fig. 1a, see Additional file 1: Table S4 and Additional file 2: Figure S1). The highest and lowest levels of introgression were identified using ESIB and EWB, respectively, as sister group. Additionally, the three populations from the producing regions in China — Duroc4, Landrace4, and Large White2 — showed the highest levels of *D*-statistic values compared to others across all three lines (Fig. 1a). When dividing the Chinese group into CEDP, CNDP, CMDP, CSWDP, and CSDP to calculate the *D*-statistic (see in Methods), all commercial lines showed the highest *D*-statistic values for CSDP (see Additional file 2: Figure S1).

To further validate the introgressed signals from Chinese pigs into ECPs, we calculated *D*-statistics also using resequencing data. Due to the insufficient number of CSWDP and CMDP samples in this dataset, we focused on CEDP, CNDP, and CSDP for our calculations. The highest *D*-statistics were observed for the CSDP group (as P3 population) compared to others (Fig. 1b and see Additional file 1: Table S5). This confirmed that the introgression from Chinese pigs is widely present in the global population of ECPs, predominantly from the CSDP. Next, utilizing SNP chip data, we estimated the admixture time between ECP and Chinese pigs (CEDP or CSDP) using ALDER software [35], which yielded a range of approximately 27.8 to 48.9 generations ago for the CSDP introgression and 26.1 to 38.0 generations for the CEDP introgression (see Additional file 2: Figure S2). Based on resequencing data, the estimated range for CSDP and CEDP introgression was 30.4 to 36.0 and 32.1 to 36.1 generations, respectively (see Additional file 2: Figure S2). Therefore, we estimate that the admixture time of ECP and Chinese pigs occurred approximately 150 to 300 years ago (based on a generation time of 5 years). The mass importation of Chinese pigs into Western countries, therefore, led to significant introgression from Chinese pigs into commercial lines since that time [4, 50].

### Genome-wide introgression from Chinese pigs into European commercial lines and selective sweep analysis

**Fig. 2:**
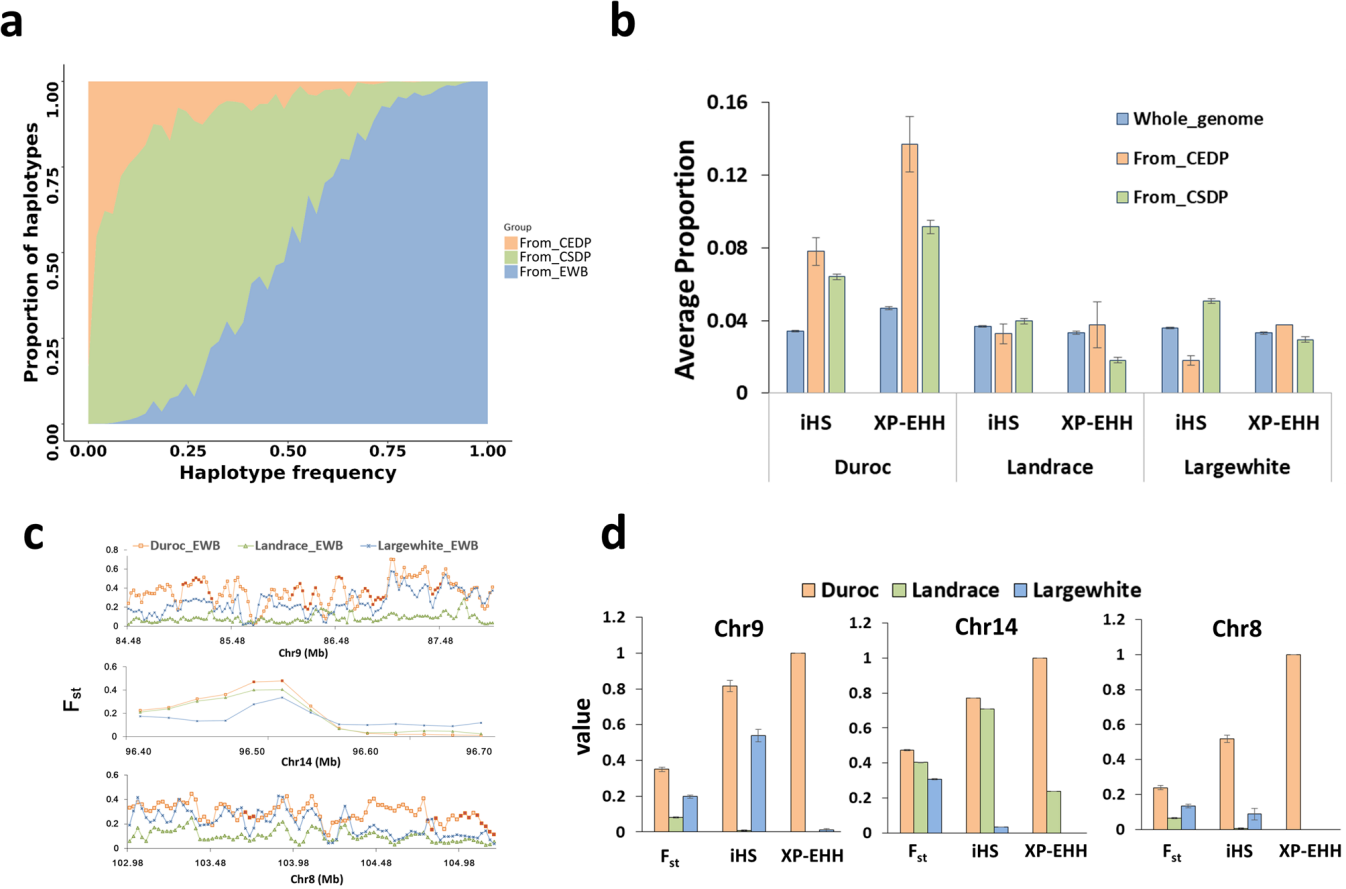
Comparisons of genome-wide introgression maps for Duroc, Landrace, and Large White pigs from CEDP and CSDP. **a** The diagram of local ancestry haplotype frequency in Duroc. The EWB, CSDP, and CEDP in the legend refer to the reference populations used for local ancestry genetic introgression detection in Duroc pigs. **b** Average proportion of SNPs with normalized |iHS score| > 2 and min score of XP-EHH > 2 within 50 Kb windows for Duroc, Landrace, and Large White. Error bars in barplots depict the standard errors of each proportion. **c** F_ST_ statistics within the regions spanning from 84.48 to 87.98 Mb on Chromosome 9, from 96.40 to 96.70 Mb on Chromosome 14 and from 102.98 to 105.00 Mb on Chromosome 8, red dots represent window bins identified as CSDP introgression. **d** The average XP-EHH, iHS and F_ST_ statistics within the positive selective regions with introgression signals described in (**c**) for Duroc, Landrace, and Large White populations. The values of iHS and XP-EHH represent the proportion of SNPs with normalized |iHS score| > 2 and a min score of XP-EHH > 2 within 50 Kb windows respectively

Because CEDP and CSDP exhibited significantly higher introgressed signals compared to CNDP (Fig. 1b), we based our analysis on the former two groups as representative Chinese pig populations contributing to the introgression into ECPs. Based on haplotype inference using the RFmix tool [36], we distinguished the 5-SNP haplotypes of ECP from three origins: EWB, CSDP, and CEDP. Frequencies of CSDP and CEDP-derived haplotypes across the whole genome ranged from 17.9 to 20.7% and 2.9 to 3.1%, respectively (Fig. 2a and see Additional file 2: Figure S3). We then defined bins (5-SNP window size) with CSDP or CEDP haplotype frequencies greater than 50% as the Chinese pig introgression regions. Although the Chinese introgressed haplotypes were distributed across the genome in the ECP populations (see Additional file 1: Table S6 and Additional file 2: Figure S4), the frequencies of these haplotypes differed among these populations (Duncan test, *p*-value < 0.001) (see Additional file 2: Figure S5). We further conducted genome-wide selective sweep analyses using iHS and XP-EHH indicators (see Additional file 1: Table S7 and S8). Compared to the genome-wide level, genomic regions that contained haplotypes introgressed from CEDP and CSDP showed a higher proportion of bins with normalized |iHS score| > 2 and minimum XP-EHH > 2 in Duroc than in Large White and Landrace (Fig. 2b). Additionally, we identified 100, 49, and 130 regions (50 Kb window size) under positive selection in the Duroc, Landrace, and Large White populations, respectively. Overlapping these regions with introgressed bins, identified 37, 1, and 19 bins in the Duroc, Landrace, and Large White populations, respectively (see Additional file 1: Table S9). The proportion of positively selected genomic bins that overlapped with Chinese introgressed haplotypes differed significantly among Duroc, Landrace, and Large White populations (*p*-value = 2.357 × 10^− 9^, *X*^2^ = 39.732, Chi-squared test). Duroc exhibited a substantially higher proportion of such enrichment (62.4%) compared to Landrace (2.0%) and Large White (14.6%), suggesting that Chinese introgressed segments in Duroc have undergone stronger positive selection than in the other two breeds. Among these Chinese introgressed haplotypes, most were identified as genetic segments derived from CSDP (see Additional file 1: Table S9). We therefore only analyzed the representative genetic segments from CSDP in subsequent analyses. The F_ST_ statistic (calculated with a 50 Kb window) was used to validate signals of local selection and identified three representative genomic regions introgressed from CSDP that exhibited higher F_ST_ values in the Duroc population compared to Large White and Landrace pigs. These regions included 84.48–87.98 Mb on Chromosome 9, 96.40–96.70 Mb on Chromosome 14, and 102.98–105.00 Mb on Chromosome 8 (Fig. 2c and d).

### CSDP and CEDP-derived haplotype-based genome-wide association study

**Fig. 3:**
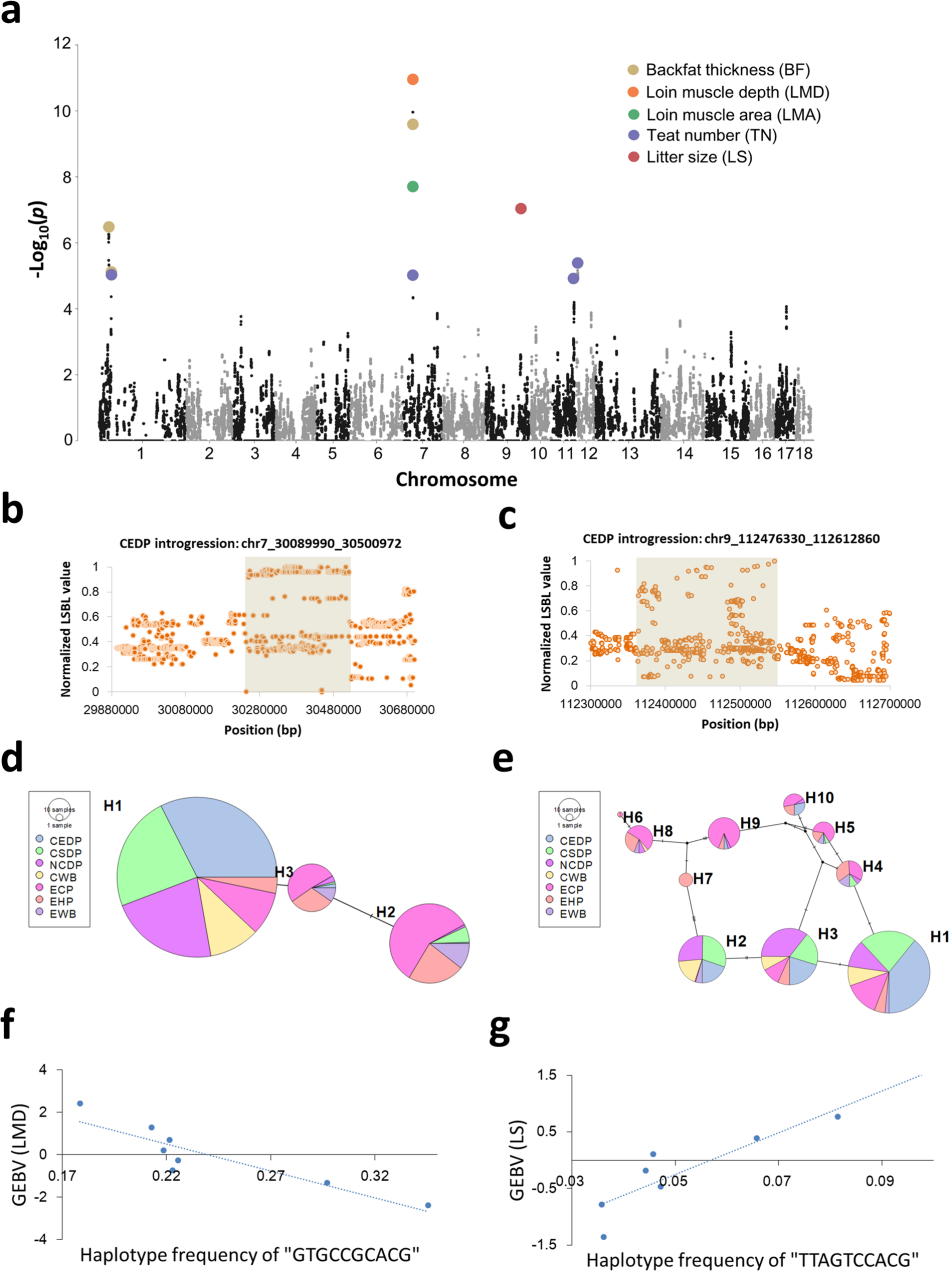
Local ancestry-derived haplotype-based genome-wide association study (GWAS) analysis. **a** Summary Manhattan plot of five phenotypes with significant signals. The most significant haplotype blocks at each quantitative trait locus (QTL) are marked with colored dots. **b** and **c** LSBL values of each SNP within the significant regions of the ancestral haplotype-based GWAS significant regions for LMD and LS traits. The shaded area denotes for the region of SNPs with the highest LSBL values. **d** and **e** The haplotype network for the 10-SNP haplotypes associated with LMD and LS traits. Each circle represents a haplotype, and the size of the circle is proportional to the haplotype frequency. The line width and length represent the differences between haplotypes. **f** and **g** The relationship between the haplotype frequency and genomic estimated breeding values (GEBVs) for LMD and LS traits. All individuals (from LCS dataset) were ranked by GEBVs and grouped into bins (*N* = 325 for each bin, depicted by each dot); the x-axis indicates the haplotype frequency within each bin, and the y-axis shows the corresponding average GEBV. The line in each plot represents the trend according to the average GEBVs in each bin

Introgression introduces substantial genetic variation, which can lead to phenotypic changes. We performed GWAS utilizing low-coverage data from 2802 Duroc pigs, based on the recoded haplotype data (see Methods). This identified 10 QTLs associated with five economic traits, with an *FDR* of less than 0.05 (see Additional file 1: Table S10 and S11). Among these, eight QTLs were identified from CEDP and two from CSDP (Fig. 3a and see Additional file 1: Table S10). Among all traits analyzed, the most significant 5-SNP haplotype was located within a QTL on Chromosome 7 associated with loin muscle depth (LMD), which was identified as influenced by genetic introgression from CEDP. This QTL was also associated with LMA and BF (see Additional file 2: Figure S6).

We computed GEBVs for each individual and selected 100 Duroc pigs with the highest and lowest GEBV for each trait (TN, LMD, LMA, BF and LS) to perform LSBL analysis to confirm the differentiation of each SNP between the high and low GEBV groups within the identified introgressed QTLs. Among the 10 QTLs, we detected significant SNP differentiation for 8 QTL regions (Fig. 3b and c and see Additional file 2: Figure S7). In each of these regions, we selected the 10 SNPs with the highest LSBL values (see Methods) and we combined them to form representative haplotypes (Fig. 3b and c), which served as markers to investigate differences among different populations utilizing the published resequencing dataset.

We also investigate the prevalence of the Chinese introgressed haplotypes and correlated with the GEBV for each trait (Fig. 3d–g and see Additional file 2: Figure S8 and S9). For the QTL associated with LMD (chr7_30089990_30500972), the haplotype network showed the H1 haplotype (“GTGCCGCACG”) to occupy the largest node (Fig. 3d), indicating its predominant status. The H1 haplotype was prevalent in the CEDP population and was also common in other Chinese populations, but was rarely found in European breeds (see Additional file 2: Figure S8), indicating the dominant H1 haplotype was derived from Chinese indigenous pigs. Notably, the frequency of the H1 haplotype decreased with an increase in GEBV for LMD trait in the Duroc pig population (Fig. 3f).

For LS, we identified a new CEDP-introgressed QTL located on Chromosome 9 that had not been identified based on SNPs (Fig. 3a and c and see Additional file 2: Figure S10). In this region, we selected the top SNPs based on LSBL values and combined them into haplotypes (Fig. 3c). The most prevalent H1 haplotype (“TTAGTCCACG”), was derived from the CEDP population and was also detected to some extent in European pigs (Fig. 3e and see Additional file 2: Figure S8). In Duroc pigs, individuals with a higher frequency of the CEDP haplotype exhibited greater LS GEBV values (Fig. 3c and see Additional file 2: Figure S8). In addition to the newly discovered QTL for LS, we identified four additional QTLs compared to the GWAS analysis using SNPs from our previous study [12], including QTLs on chromosome 1 for BF, on chromosomes 1 and 7 for TN, and on chromosome 7 for LMA (Fig. 3a and see Additional file 1: Table S10). Through further comparative analysis of LSBL values, we identified representative haplotypes from Chinese pig populations, which were designated as H1 haplotypes (see Additional file 2: Figures S7 and S9). These included “TCGCATGCCC” for TN and “TCTTGGGCAT” for BF from the CSDP population, as well as “CCGCGCGCGT” for TN, “CTGGACCTTT” for LMA, and “CAAGAGCCCT” and “CCGGGATTAT” for BF from the CEDP population (see Additional file 2: Figure S8). Correlation analyses between the frequencies of these H1 haplotypes and the GEBVs of corresponding traits revealed negative associations, suggesting that these introgressed haplotypes may have negative effects on economic performance (see Additional file 2: Figure S11).

### CSDP and CEDP introgression in relation to transcriptome expression profile

**Fig. 4:**
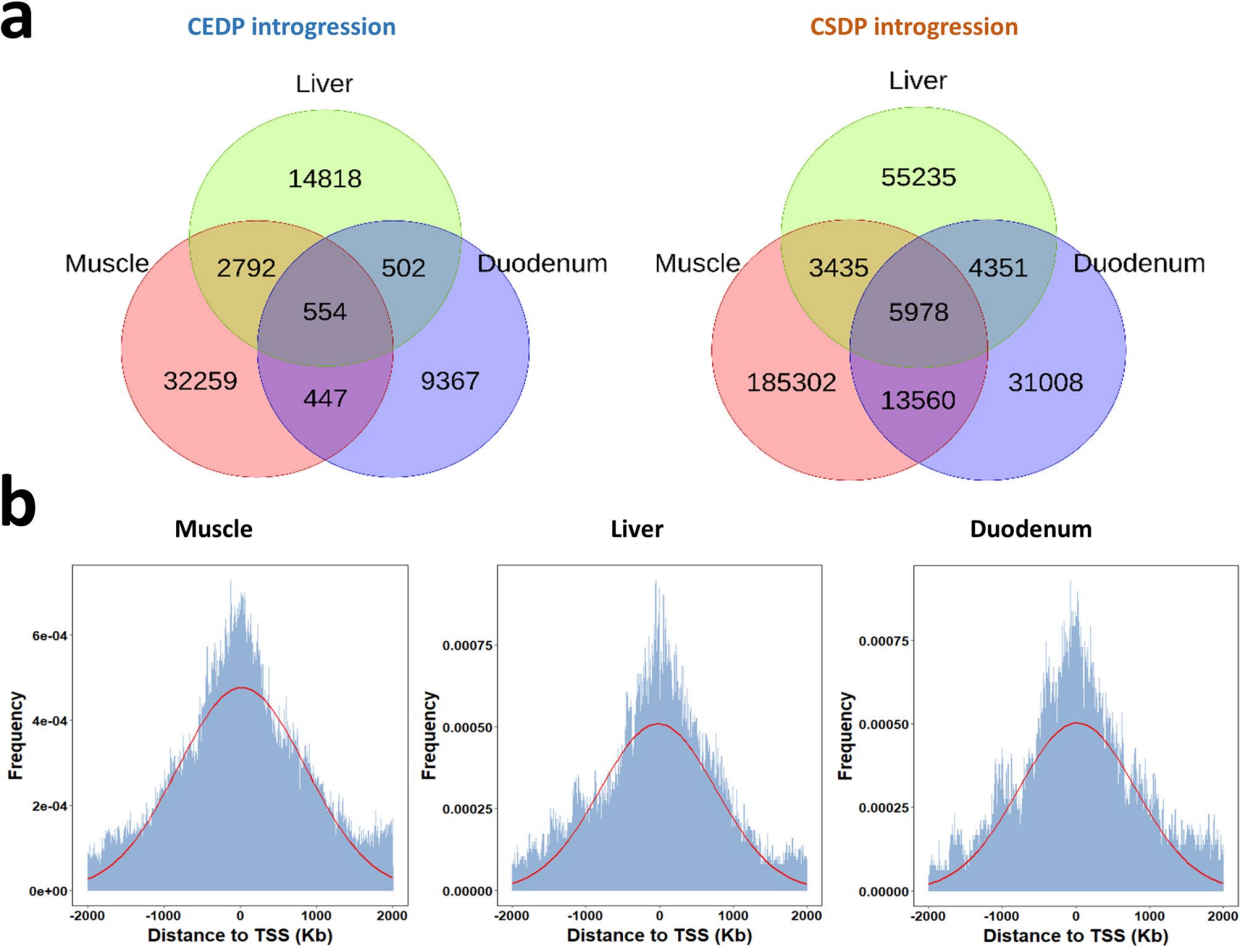
Expression genomew-wide associations study (eGWAS) analysis based on Chinese ancestral haplotypes derived from the CEDP and CSDP within the Duroc population. **a** Venn diagrams showing shared and unique haplotypes derived from CEDP and CSDP that are significantly associated with gene expression across muscle, liver, and duodenum tissues. **b** Density plots (red curves) showing the distribution of distances between significantly associated introgressed haplotype blocks (based on combined data from both CEDP and CSDP populations) and the nearest transcription start sites (TSS) in muscle, liver, and duodenum tissues

Genetic introgression likely has a direct impact on gene expression profiles, potentially resulting in phenotypic changes. To investigate this, we conducted an eGWAS analysis using the presence or absence of Chinese ancestral haplotypes as explanatory variables in 100 Duroc pigs and theirtranscriptome data from muscle (*N* = 97), liver (*N* = 92), and duodenum tissues (*N* = 96). We identified 36,052, 18,666, and 10,870 bins originated from CEDP population that were significantly associated with the expression of 64, 24, and 21 genes in muscle, liver, and duodenum tissues, respectively (Bonferroni corrected *p*-value < 0.05) (Fig. 4a and see Additional file 1: Table S12). For CSDP introgressed bins, 208,275, 68,999, and 54,897 bins were significantly associated with the expression of 286, 100, and 89 genes in muscle, liver, and duodenum tissues, respectively (Bonferroni corrected *p*-value < 0.05) (Fig. 4a and see Additional file 1: Table S12). Among these, only a small number of bins overlapped across the three tissues, with the majority displaying tissue-specific signals. The proportions of tissue-specific signals for CEDP introgression in muscle, liver, and duodenum were 89.5, 79.4, and 86.7%, respectively, while for CSDP introgression, these figures were 89.0, 80.1, and 56.5%. Examination of the distribution of bins based on their proximity to the nearest transcription start site (TSS) indicated that most significant introgressed bins that associated with gene expression were located near their respective TSS across all three tissues. This was shown based on analysis of both the combined data from the CEDP and CSDP populations (Fig. 4b) and of their separate data (see Additional file 2: Figure S12).

### Colocalization of loci associated with loin muscle depth and TAF11 expression

**Fig. 5:**
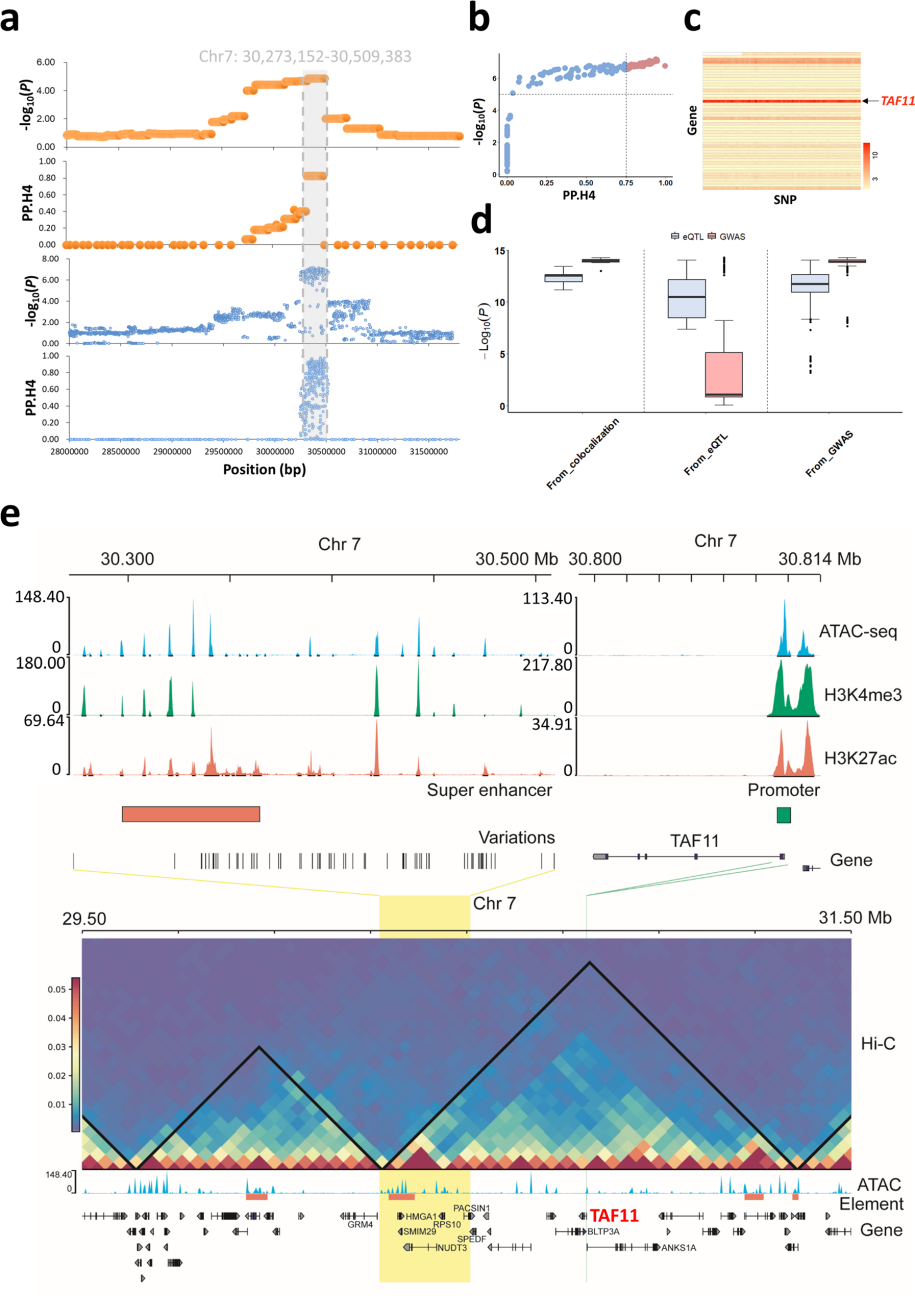
Co-localization analysis of muscle traits within the Duroc population. **a** The co-localization analysis integrates GWAS and eGWAS signals using the coloc R package and SMR tool. The plots featuring orange are based on the CEDP-derived haplotype blocks, while the blue ones are based on SNPs. **b** The plot of coloc and SMR results based on SNPs, with the x-axis and y-axis representing the results of coloc and SMR analyses respectively. The red dots represent the top 73 SNP sites that are significant in both coloc (PP.H4 > 0.75) and SMR (*p*-value < 1e-5) results. **c** The association of the top 73 significant SNPs with gene expression within the genomic region of 28–32 Mb, where the x-axis represents SNPs and the y-axis represents genes, with the heat value representing the -log10 value of significance (*p*-value) in eGWAS analysis. **d** The distribution of SNP significance (*p*-value) based on co-localization, eGWAS, and GWAS analyses, the *p*-value of each SNP was according to eGWAS (blue boxplot) and GWAS (red boxplot) results respectively, the horizontal line in each boxplot depicts the median value. **e** The upper panel displays the peak calls from ATAC-seq and ChIP-seq (Duroc samples) in the co-localized region and surrounding the *TAF11* region on Chromosome 7. The red and green boxes represent the annotated super-enhancer and promoter region of *TAF11*, respectively. The lower panel depicts the Hi-C chromatin map in co-localization and *TAF11* regions, and both regions are within the same topologically associating domain (the black triangle). The yellow region represents the region of the 73 significant SNPs

Based on the previous analysis, we identified the most significant GWAS signals for CEDP introgression of LMD compared to other traits and we, therefore, focused on these signals and combined them with eGWAS signals to conduct a co-localization analysis. Based on the results of the GWAS analysis of CEDP introgression, conducted using the coloc R package and the SMR method [45, 46], we identified the most significant signals within the region from 30.3 to 30.6 Mb on chromosome 7 (Fig. 5a and see Additional file 1: Table S13 and S14). To validate these findings, we performed the same analysis using SNPs as markers, which revealed the most significant SNPs within the same region (Fig. 5a). Combined these results using the coloc R package and the SMR method identified 73 top significant SNPs as candidate causative mutations (PP.H4 > 0.75 from coloc and *p*-value < 1e-5 from SMR) (Fig. 5b and see Additional file 1: Table S15). Among these 73 SNPs, the strongest association with gene expression was observed for the *TAF11* gene (Fig. 5c). Moreover, we compared the significance levels (–log_₁₀_(p)) of SNPs from three distinct sources to assess the strength of association signals (Fig. 5d). The first group (“From_colocalization”) included the 73 co-localized SNPs, representing co-localized variants associated with both *TAF11* expression and LMD. The second group (“From_eQTL”) consisted of SNPs significantly associated with *TAF11* expression from eQTL analysis. And the third group (“From_GWAS”) included SNPs from the 30.3–30.6 Mb region identified by GWAS for LMD. For each group, we extracted the *p*-values from both GWAS and eQTL analyses and compared the significance based on significant SNPs detected from GWAS, eGWAS and co-localized analysis. As shown in Fig. 5d showed, the co-localized SNPs showed the strongest and most consistent significance across both analyses, highlighting their potential as functional candidates. We therefore defined these 73 SNPs as the most likely candidate loci associated with both *TAF11* expression and LMD phenotype.

A maximum likelihood phylogenetic tree based on these 73 SNPs revealed that Duroc pigs with the highest LMD GEBV (DUROC-h) clustered closely with EWB, whereas those with the lowest LMD GEBV (DUROC-l) were more closely related to the Chinese pig group (see Additional file 2: Figure S13). The haplotype heatmap (see Additional file 2: Figure S13), further supported that this 73-SNP genomic segment was introgressed from Chinese pigs.

We further analyzed epigenomic data to identify potential regulatory factors linking this region containing the 73 SNPs to *TAF11* gene expression. We characterized the epigenetic modification signals within this region and identified a high density of open chromatin regions (OCRs), suggesting the presence of potential cis-regulatory elements that regulate related genes in muscle tissue (Fig. 5e and see Additional file 2: Figure S14). We also observed that these OCRs were heavily marked by H3K27ac histone modifications, with high H3K27ac signal peaks clustering in this region compared to surrounding areas. In addition, a super-enhancer of approximately 67.40 kb in size was identified within this region (Chr7: 30,297,170 − 30,364,571 bp) (Fig. 5e and see Additional file 2: Figure S14). The promoter region of *TAF11* was enriched with ATAC-seq and H3K4me3 signal peaks, indicating that the chromatin in this region was open and there includes epigenetic modifications associated with active transcription (Fig. 5e and see Additional file 2: Figure S14). Based on Hi-C chromatin interaction data, we discovered that the super-enhancer and *TAF11* are located within the same TAD domain (Fig. 5e). These findings further reinforce the evidence supporting a regulatory relationship between the CEDP introgressed region and *TAF11* gene expression, which likely contribute to variation in LMD in Duroc pigs.

## Discussion

### Genetic signatures of the introgression from Chinese pigs in commercial European lines

In our study, we identified that introgression from Chinese pigs is widespread across European commercial pig populations, rather than being restricted to a single or limited group (Fig. 1 and see Additional file 2: Figure S1). We estimated that these hybridization events between Chinese and European pigs occurred approximately 150 to 300 years ago (see Additional file 2: Figure S2), which is consistent with previous reports [4, 50]. This widespread historical introgression likely reflects the substantial genetic contribution of Chinese pigs to the formation and improvement of modern ECP.

According to the *D*-statistic results based on the SNP chip data (see Additional file 1: Table S4), we identified relatively higher *D*-statistics in Duroc, Landrace, and Large White pigs from the producing regions in China compared to others (Duroc4, Landrace4, and Large White2) (Fig. 1a), which suggested that more hybridization events with Chinese pigs may have occurred in the history of these populations since their importation to China. Among the Chinese pig populations, CSDP contributed the most to the introgression into European pigs compared to other Chinese indigenous groups (see Additional file 2: Figure S1), but other populations also made significant contributions to the introgression into ECP lines, such as CMDP, CSWDP, and CEDP. An unexpected finding was that a higher *D*-statistic was observed when using ESIB and EHUMA as the sister group (P1) compared to EWB (Fig. 1a), which may be attributed to two primary reasons: (1) The reference genome utilized in this study was derived from a Duroc pig, which may inflate the shared derived alleles between the test population and reference-related populations; (2) According to Yang et al. [9], the EWB samples represent a geographically diverse panel of wild boars in Europe, some of which exhibit detectable Asian genetic components. This admixture likely increased the number of shared alleles between EWB and Chinese pigs, leading to an elevated BABA count and consequently reducing the *D*-statistic when EWB was used as the P1 population. In contrast, ESIB and EHUMA are European local breeds with limited historical gene flow from Asian pigs [9] and, therefore, produced higher *D*-statistics when designated as the sister group. Consistent with this, our results showed that the average BABA value was highest when EWB (2953.672) served as P1, compared to ESIB (2549.541) and EHUMA (2681.241) (see Additional file 1: Table S4). This interpretation also is supported by our F_ST_ analysis, which revealed significantly higher genetic differentiation in the comparisons between Chinese pigs (CSDP or CEDP) and EDP (ESIB or EHUMA) than between Chinese pigs and EWB (*p*-value < 0.05, Duncan’s test) (see Additional file 2: Figure S15), further supporting the reduced allele sharing between Chinese pigs and the EDP group. For the resequencing data analyses, these groups were excluded from further local introgression analyses due to the limited sample sizes of the CSWDP and CMDP populations. Future studies with larger sample sizes may be necessary for a more comprehensive investigation of multi-ancestry contributions to ECP lines.

The Duroc pig is often used as the terminal sire in three-way crossbreeding systems, thereby experiencing the highest selective pressure compared to Landrace and Large White pigs [1]. We found that the Chinese-derived genetic segments underwent stronger selection in Duroc pigs than in Landrace and Large White pigs. Some of the genes in these regions may be under positive selection, such as the *AHR* gene on chromosome 9, which has been found to be associated with fertility and meat production traits in pigs [4, 51]. This positive selection may be attributed to human preferences in pig production, specifically the emphasis on increasing litter size and enhancing meat yield within the pig industry. Furthermore, we found that the frequencies of haplotypes derived from the CEDP and CSDP in EDLY and EWDU pigs were more closely correlated with those in Duroc than in Landrace and Large White pigs (see Additional file 2: Figure S16 and S17). Altogether these results suggest higher selective pressure in the Duroc breed compared to Landrace and Large White pigs, which could have led to the introgression of genomic segments from Chinese pigs into crossbred pigs predominantly being contributed via Duroc.

### Chinese-derived haplotypes played a key role in the performance of economic trait and gene expression profile in Duroc pigs

Previous studies have illustrated the effects of introgression from Chinese pigs into European breeds and have identified several key loci that show evidence of introgression, including in the *AHR*, *KATNAL1*, and *NDUFS4* genes, which are associated with sow fertility [3, 4, 50, 52], the *GOLM1-NAA35* locus, which is linked to enhanced disease resistance [50], the *EDNRB* variant, which is associated with coat color [6], and the *LEMD3* gene, which is related to ear morphology [6]. We performed an ancestral haplotype-based GWAS analysis across the whole genome and identified several CSDP and CEDP introgressed regions associated with economic traits. Notably, five of these overlapped with regions identified in our previous research [12] (Fig. 3a and see Additional file 1: Table S10). In addition to these QTLs, we also identified five novel QTLs (see Additional file 1: Table S10), which may represent novel genetic loci associated with their corresponding traits. For example, the QTL associated with litter size (LS) was identified in the intergenic region 112,476,330 − 112,612,860 bp region on chromosome 9 (Fig. 3b and see Additional file 1: Table S10). The nearest genes, *TPK1* and *NOBOX*, were both highly expressed in the oocytes of pigs, with *NOBOX* being well-known for its role in ovarian differentiation and folliculogenesis [53–55]. In addition, the QTL associated with loin muscle area (LMA) is the same as that for LMD and BF, indicating that this QTL regulates both muscle growth and fat deposition, although the underlying regulatory mechanisms require further investigation. Other newly discovered QTLs include two associated with teat number located on chromosome 7 (29,416,728 − 30,081,172 bp) and on chromosome 1 (38,528,791 − 39,201,240 bp), as well as one associated with BF on chromosome (38,063,399 − 39,118,175 bp). These QTLs exhibited lower significance compared to those identified for LMD, LMA, and LS, and multiple genes were present within these regions (see Additional file 1: Table S10), suggesting that the introgression from Chinese pigs may influence a complex gene regulatory network, leading to increased variation in economic traits. However, we did not identify the QTL on chromosome 7 with a major effect associated with teat number (TN) that was reported in the previous study [12], indicating that this major QTL was not introduced by CEDP and CSDP introgression.

Gene expression profile differ between pig populations [56]. We utilized transcriptome data from three tissues and the introgressed haplotypes from Chinese pigs to conduct an eGWAS analysis. The significant haplotypes identified were predominantly enriched near the transcript start site (TSS) (Fig. 4b), suggesting that variation resulting from introgression may influence gene regulation. In line with this, a previous study demonstrated that alleles from different pig breeds can affect the transcriptome in crossbred offspring [57]. Additionally, multiple ancestral haplotypes may harbor regulatory mutations that cumulatively contribute to a QTL that affects complex traits [58]. Therefore, the Chinese introgression in the Duroc breed that originates from distinct population groups, such as CEDP and CSDP, may lead to a broader range of gene expression variation by introducing allele-specific variants. A broader tissue expression profile and larger sample size are necessary for further investigation into the effects of multiple ancestral introgressed haplotypes.

### TAF11 expression and Chinese-derived haplotypes, and their association to muscle growth trait in Duroc pigs

*TAF11* is a gene that encodes a subunit of the transcription factor IID (TFIID) complex, which plays a critical role in regulating gene expression [59]. Previous studies have illustrated its association with muscle growth traits, such as LMA and LMD [26, 60]. Li et al. discovered a variant located in the promoter region of *TAF11* that is associated with both *TAF11* expression and the LMD phenotype [26]. They speculated that there may be additional chromatin interactions involved in the transcriptional regulation of *TAF11*. We focused on LMD and performed GWAS analysis based on CEDP-derived haplotype blocks, and the result exhibited more significant association signals of the introgressed haplotypes with LMD compared to other traits. Based on introgressed haplotype blocks and SNPs, we conducted co-localization analysis and confirmed the association of the introgression region on chromosome 7 with *TAF11* expression and LMD variation (Fig. 5a–d), despite the introgressed region being more than 300 Kb away from the *TAF11* promoter. By integrating Hi-C, ATAC-seq, H3K4me3, and H3K27ac signal peaks, our results suggest that regulatory variants in the introgressed region modulate *TAF11* gene expression through a super-enhancer and its associated chromatin interactions within a TAD domain (Fig. 5e), ultimately contributing to phenotypic variation for LMD in the Duroc population. Further exploration is needed to elucidate the molecular mechanisms underlying the remote regulation of *TAF11* expression due to this introgression region.

## Conclusions

This study demonstrates the genetic signatures of haplotypes introgressed from Chinese pigs and highlights their significant contribution to economically important traits in the Duroc breed. Through GWAS analysis based on Chinese-derived haplotypes, we identified five novel QTLs and confirmed five previously identified QTL. The eGWAS analysis revealed that Chinese introgression enhanced expression diversity within the Duroc population. Notably, the co-localization of GWAS and eGWAS signals identified a super-enhancer located approximately 300 Kb from *TAF11*, within the same topologically associating domain, which is associated with loin muscle depth and *TAF11* expression in muscle tissue. These findings provide valuable insights into the genetic and molecular impacts of introgression from Chinese pigs on economically important traits and offer a potential foundation for future genomic breeding studies related to Chinese-derived haplotypes in pig breeding.

## Supplementary Information

Below is the link to the electronic supplementary material.

Supplementary Material 1

Supplementary Material 2

## Data Availability

All data and materials were collected from previously published studies.

## Declarations

### Benefit-sharing statement

The benefits of this research accrue from the sharing of our data and results in public databases as described above.

## References

[1] Wang L, Wang A, Wang L, Li K, Yang G, He R, et al. Animal genetic resources in China pigs. Beijing: China Agricultural; 2011.

[2] Groenen MA, Archibald AL, Uenishi H, Tuggle CK, Takeuchi Y, Rothschild MF, et al. Analyses of pig genomes provide insight into Porcine demography and evolution. Nature. 2012;491:393–8. 23151582 10.1038/nature11622 PMC3566564

[3] Bosse M, Lopes MS, Madsen O, Megens HJ, Crooijmans RP, Frantz LA, et al. Artificial selection on introduced Asian haplotypes shaped the genetic architecture in European commercial pigs. Proc Biol Sci. 2015;282:20152019. 26702043 10.1098/rspb.2015.2019 PMC4707752

[4] Bosse M, Megens HJ, Frantz LA, Madsen O, Larson G, Paudel Y, et al. Genomic analysis reveals selection for Asian genes in European pigs following human-mediated introgression. Nat Commun. 2014;5:4392. 25025832 10.1038/ncomms5392 PMC4225517

[5] Frantz LA, Schraiber JG, Madsen O, Megens HJ, Cagan A, Bosse M, et al. Evidence of long-term gene flow and selection during domestication from analyses of Eurasian wild and domestic pig genomes. Nat Genet. 2015;47:1141–8. 26323058 10.1038/ng.3394

[6] Wilkinson S, Lu ZH, Megens HJ, Archibald AL, Haley C, Jackson IJ, et al. Signatures of diversifying selection in European pig breeds. PLoS Genet. 2013;9:e1003453. 23637623 10.1371/journal.pgen.1003453 PMC3636142

[7] Yang J, Huang L, Yang M, Fan Y, Li L, Fang S, et al. Possible introgression of the VRTN mutation increasing vertebral number, carcass length and teat number from Chinese pigs into European pigs. Sci Rep. 2016;6:19240. 26781738 10.1038/srep19240 PMC4726066

[8] Chen M, Su G, Fu J, Wang A, Liu JF, Lund MS, et al. Introgression of Chinese haplotypes contributed to the improvement of Danish duroc pigs. Evol Appl. 2019;12:292–300. 30697340 10.1111/eva.12716 PMC6346729

[9] Yang B, Cui L, Perez-Enciso M, Traspov A, Crooijmans R, Zinovieva N, et al. Genome-wide SNP data unveils the globalization of domesticated pigs. Genet Sel Evol. 2017;49:71. 28934946 10.1186/s12711-017-0345-y PMC5609043

[10] Wang Y, Zhang C, Peng Y, Cai X, Hu X, Bosse M, et al. Whole-genome analysis reveals the hybrid formation of Chinese Indigenous DHB pig following human migration. Evol Appl. 2022;15:501–14. 35386394 10.1111/eva.13366 PMC8965386

[11] Tong X, Chen D, Hu J, Lin S, Ling Z, Ai H, et al. Accurate haplotype construction and detection of selection signatures enabled by high quality pig genome sequences. Nat Commun. 2023;14:5126. 37612277 10.1038/s41467-023-40434-3 PMC10447580

[12] Yang R, Guo X, Zhu D, Tan C, Bian C, Ren J, et al. Accelerated Deciphering of the genetic architecture of agricultural economic traits in pigs using a low-coverage whole-genome sequencing strategy. Gigascience. 2021;10:giab048. 34282453 10.1093/gigascience/giab048 PMC8290195

[13] Tan C, Wu Z, Ren J, Huang Z, Liu D, He X, et al. Genome-wide association study and accuracy of genomic prediction for teat number in duroc pigs using genotyping-by-sequencing. Genet Sel Evol. 2017;49:35. 28356075 10.1186/s12711-017-0311-8 PMC5371258

[14] Bian C, Prakapenka D, Tan C, Yang R, Zhu D, Guo X, et al. Haplotype genomic prediction of phenotypic values based on chromosome distance and gene boundaries using low-coverage sequencing in duroc pigs. Genet Sel Evol. 2021;53:78. 34620094 10.1186/s12711-021-00661-y PMC8496108

[15] Crespo-Piazuelo D, Acloque H, Gonzalez-Rodriguez O, Mongellaz M, Mercat MJ, Bink M, et al. Identification of transcriptional regulatory variants in pig duodenum, liver, and muscle tissues. Gigascience. 2022;12:giad042. 37354463 10.1093/gigascience/giad042 PMC10290502

[16] Zhao Y, Hou Y, Xu Y, Luan Y, Zhou H, Qi X, et al. A compendium and comparative epigenomics analysis of cis-regulatory elements in the pig genome. Nat Commun. 2021;12:2217. 33850120 10.1038/s41467-021-22448-x PMC8044108

[17] Danecek P, Auton A, Abecasis G, Albers CA, Banks E, DePristo MA, et al. The variant call format and vcftools. Bioinformatics. 2011;27:2156–8. 21653522 10.1093/bioinformatics/btr330 PMC3137218

[18] Xing Y, Li G, Wang Z, Feng B, Song Z, Wu C. GTZ: a fast compression and cloud transmission tool optimized for FASTQ files. BMC Bioinformatics. 2017;18:549. 29297296 10.1186/s12859-017-1973-5 PMC5751770

[19] Browning BL, Zhou Y, Browning SR. A one-penny imputed genome from next-generation reference panels. Am J Hum Genet. 2018;103:338–48. 30100085 10.1016/j.ajhg.2018.07.015 PMC6128308

[20] Narasimhan V, Danecek P, Scally A, Xue Y, Tyler-Smith C, Durbin R. BCFtools/RoH: a hidden Markov model approach for detecting autozygosity from next-generation sequencing data. Bioinformatics. 2016;32:1749–51. 26826718 10.1093/bioinformatics/btw044 PMC4892413

[21] Bolger AM, Lohse M, Usadel B. Trimmomatic: a flexible trimmer for illumina sequence data. Bioinformatics. 2014;30:2114–20. 24695404 10.1093/bioinformatics/btu170 PMC4103590

[22] Langmead B, Trapnell C, Pop M, Salzberg SL. Ultrafast and memory-efficient alignment of short DNA sequences to the human genome. Genome Biol. 2009;10:R25. 19261174 10.1186/gb-2009-10-3-r25 PMC2690996

[23] Danecek P, Bonfield JK, Liddle J, Marshall J, Ohan V, Pollard MO, et al. Twelve years of samtools and BCFtools. Gigascience. 2021;10:giab008. 33590861 10.1093/gigascience/giab008 PMC7931819

[24] Kim D, Paggi JM, Park C, Bennett C, Salzberg SL. Graph-based genome alignment and genotyping with HISAT2 and HISAT-genotype. Nat Biotechnol. 2019;37:907–15. 31375807 10.1038/s41587-019-0201-4 PMC7605509

[25] Pertea M, Pertea GM, Antonescu CM, Chang TC, Mendell JT, Salzberg SL. StringTie enables improved reconstruction of a transcriptome from RNA-seq reads. Nat Biotechnol. 2015;33:290–5. 25690850 10.1038/nbt.3122 PMC4643835

[26] Li JJ, Xiang Y, Zhang L, Qi XL, Zheng ZQ, Zhou P, et al. Enhancer-promoter interaction maps provide insights into skeletal muscle-related traits in pig genome. BMC Biol. 2022;20:136. 35681201 10.1186/s12915-022-01322-2 PMC9185926

[27] Li H, Durbin R. Fast and accurate short read alignment with Burrows-Wheeler transform. Bioinformatics. 2009;25:1754–60. 19451168 10.1093/bioinformatics/btp324 PMC2705234

[28] Heinz S, Benner C, Spann N, Bertolino E, Lin YC, Laslo P, et al. Simple combinations of lineage-determining transcription factors prime cis-regulatory elements required for macrophage and B cell identities. Mol Cell. 2010;38:576–89. 20513432 10.1016/j.molcel.2010.05.004 PMC2898526

[29] Hnisz D, Abraham BJ, Lee TI, Lau A, Saint-Andre V, Sigova AA, et al. Super-enhancers in the control of cell identity and disease. Cell. 2013;155:934–47. 24119843 10.1016/j.cell.2013.09.053 PMC3841062

[30] Chen S, Zhou Y, Chen Y, Gu J. Fastp: an ultra-fast all-in-one FASTQ preprocessor. Bioinformatics. 2018;34:i884–90. 30423086 10.1093/bioinformatics/bty560 PMC6129281

[31] Abdennur N, Fudenberg G, Flyamer IM, Galitsyna AA, Goloborodko A, Imakaev M, et al. Pairtools: from sequencing data to chromosome contacts. Plos Comput Biol. 2024;20:e1012164. 38809952 10.1371/journal.pcbi.1012164 PMC11164360

[32] Abdennur N, Mirny LA. Cooler: scalable storage for Hi-C data and other genomically labeled arrays. Bioinformatics. 2020;36:311–6. 31290943 10.1093/bioinformatics/btz540 PMC8205516

[33] Durand NC, Shamim MS, Machol I, Rao SS, Huntley MH, Lander ES, et al. Juicer provides a one-click system for analyzing loop-resolution Hi-C experiments. Cell Syst. 2016;3:95–8. 27467249 10.1016/j.cels.2016.07.002 PMC5846465

[34] Malinsky M, Matschiner M, Svardal H. Dsuite - Fast D-statistics and related admixture evidence from VCF files. Mol Ecol Resour. 2021;21:584–95. 33012121 10.1111/1755-0998.13265 PMC7116594

[35] Loh PR, Lipson M, Patterson N, Moorjani P, Pickrell JK, Reich D, et al. Inferring admixture histories of human populations using linkage disequilibrium. Genetics. 2013;193:1233–54. 23410830 10.1534/genetics.112.147330 PMC3606100

[36] Maples BK, Gravel S, Kenny EE, Bustamante CD. RFMix: a discriminative modeling approach for rapid and robust local-ancestry inference. Am J Hum Genet. 2013;93:278–88. 23910464 10.1016/j.ajhg.2013.06.020 PMC3738819

[37] Atkinson EG, Maihofer AX, Kanai M, Martin AR, Karczewski KJ, Santoro ML, et al. Tractor uses local ancestry to enable the inclusion of admixed individuals in GWAS and to boost power. Nat Genet. 2021;53:195–204. 33462486 10.1038/s41588-020-00766-y PMC7867648

[38] Bu L, Wang Y, Tan L, Wen Z, Hu X, Zhang Z, et al. Haplotype analysis incorporating ancestral origins identified novel genetic loci associated with chicken body weight using an advanced intercross line. Genet Sel Evol. 2024;56:78. 39707197 10.1186/s12711-024-00946-y PMC11660596

[39] Yang J, Lee SH, Goddard ME, Visscher PM. GCTA: a tool for genome-wide complex trait analysis. Am J Hum Genet. 2011;88:76–82. 21167468 10.1016/j.ajhg.2010.11.011 PMC3014363

[40] Strimmer K. Fdrtool: a versatile R package for estimating local and tail area-based false discovery rates. Bioinformatics. 2008;24:1461–2. 18441000 10.1093/bioinformatics/btn209

[41] Wang C, Prakapenka D, Wang S, Pulugurta S, Runesha HB, Da Y. GVCBLUP: a computer package for genomic prediction and variance component estimation of additive and dominance effects. BMC Bioinformatics. 2014;15:270. 25107495 10.1186/1471-2105-15-270 PMC4133608

[42] Shriver MD, Kennedy GC, Parra EJ, Lawson HA, Sonpar V, Huang J, et al. The genomic distribution of population substructure in four populations using 8,525 autosomal SNPs. Hum Genomics. 2004;1:274–86. 15588487 10.1186/1479-7364-1-4-274 PMC3525267

[43] Leigh JW, Bryant D. POPART: full-feature software for haplotype network construction. Methods Ecol Evol. 2015;6:1110–6.

[44] Ongen H, Buil A, Brown AA, Dermitzakis ET, Delaneau O. Fast and efficient QTL mapper for thousands of molecular phenotypes. Bioinformatics. 2016;32:1479–85. 26708335 10.1093/bioinformatics/btv722 PMC4866519

[45] Wang G, Sarkar A, Carbonetto P, Stephens M. A simple new approach to variable selection in regression, with application to genetic fine mapping. J R Stat Soc Ser B Stat Methodol. 2020;82:1273–300. 10.1111/rssb.12388 PMC10201948 37220626

[46] Zhu Z, Zhang F, Hu H, Bakshi A, Robinson MR, Powell JE, et al. Integration of summary data from GWAS and eQTL studies predicts complex trait gene targets. Nat Genet. 2016;48:481–7. 27019110 10.1038/ng.3538

[47] Purcell S, Neale B, Todd-Brown K, Thomas L, Ferreira MA, Bender D, et al. PLINK: a tool set for whole-genome association and population-based linkage analyses. Am J Hum Genet. 2007;81:559–75. 17701901 10.1086/519795 PMC1950838

[48] Pickrell JK, Pritchard JK. Inference of population splits and mixtures from genome-wide allele frequency data. PLoS Genet. 2012;8:e1002967. 23166502 10.1371/journal.pgen.1002967 PMC3499260

[49] Ramirez F, Bhardwaj V, Arrigoni L, Lam KC, Gruning BA, Villaveces J, et al. High-resolution tads reveal DNA sequences underlying genome organization in flies. Nat Commun. 2018;9:189. 29335486 10.1038/s41467-017-02525-w PMC5768762

[50] Chen H, Huang M, Yang B, Wu Z, Deng Z, Hou Y, et al. Introgression of Eastern Chinese and Southern Chinese haplotypes contributes to the improvement of fertility and immunity in European modern pigs. Gigascience. 2020;9:giaa014. 32141510 10.1093/gigascience/giaa014 PMC7059266

[51] Hou RD, Chen L, Liu XC, Liu H, Shi GH, Hou XH, et al. Integrating genome-wide association study with RNA-sequencing reveals 9 as a candidate gene influencing loin muscle area in Beijing black pigs. Biology (Basel). 2022;11:1635. 36358336 10.3390/biology11111635 PMC9687167

[52] Liu C, Huang R, Su G, Hou L, Zhou W, Liu Q, et al. Introgression of pigs in Taihu lake region possibly contributed to the improvement of fertility in Danish large white pigs. BMC Genomics. 2023;24:733. 38049711 10.1186/s12864-023-09860-x PMC10694980

[53] Huang JJ, Ruan Y, Xiao MM, Dai LG, Jiang CM, Li JF, et al. Association between polymorphisms in and litter size traits in Xiangsu pigs. Front Vet Sci. 2024;11:359312. 10.3389/fvets.2024.1359312 PMC10959092 38523712

[54] Rajkovic A, Pangas SA, Ballow D, Suzumori N, Matzuk MM. NOBOX deficiency disrupts early folliculogenesis and oocyte-specific gene expression. Science. 2004;305:1157–9. 15326356 10.1126/science.1099755

[55] Wu K, Zhai Y, Qin M, Zhao C, Ai N, He J, et al. Genetic evidence for differential functions of Figla and nobox in zebrafish ovarian differentiation and folliculogenesis. Commun Biol. 2023;6:1185. 37990081 10.1038/s42003-023-05551-1 PMC10663522

[56] Li Q, Hao M, Zhu J, Yi L, Cheng W, Xie Y, et al. Comparison of differentially expressed genes in longissimus dorsi muscle of Diannan small ears, Wujin and landrace pigs using RNA-seq. Front Vet Sci. 2023;10:1296208. 38249550 10.3389/fvets.2023.1296208 PMC10796741

[57] Lin Y, Li J, Chen L, Bai J, Zhang J, Wang Y, et al. Allele-specific regulatory effects on the pig transcriptome. Gigascience. 2022;12:giad076. 37776365 10.1093/gigascience/giad076 PMC10541795

[58] Wang Y, Cao X, Luo C, Sheng Z, Zhang C, Bian C, et al. Multiple ancestral haplotypes harboring regulatory mutations cumulatively contribute to a QTL affecting chicken growth traits. Commun Biol. 2020;3:472. 32859973 10.1038/s42003-020-01199-3 PMC7455696

[59] Gupta K, Watson AA, Baptista T, Scheer E, Chambers AL, Koehler C, et al. Architecture of TAF11/TAF13/TBP complex suggests novel regulation properties of general transcription factor TFIID. Elife. 2017;6:e30395. 29111974 10.7554/eLife.30395 PMC5690282

[60] Zhuang Z, Li S, Ding R, Yang M, Zheng E, Yang H, et al. Meta-analysis of genome-wide association studies for loin muscle area and loin muscle depth in two duroc pig populations. PLoS ONE. 2019;14:e0218263. 31188900 10.1371/journal.pone.0218263 PMC6561594

