# Supplementary material for: The impact of haplotypes derived from Chinese pigs on genetic variation and economic traits in the Duroc breed
Source: Genet Sel Evol. 2025 Oct 23;57:58. doi: 10.1186/s12711-025-01010-z (PMC12551222; doi:10.1186/s12711-025-01010-z)
Supplement: Supplementary file 2 — Supplementary Material 2 [file 12711_2025_1010_MOESM2_ESM.docx]

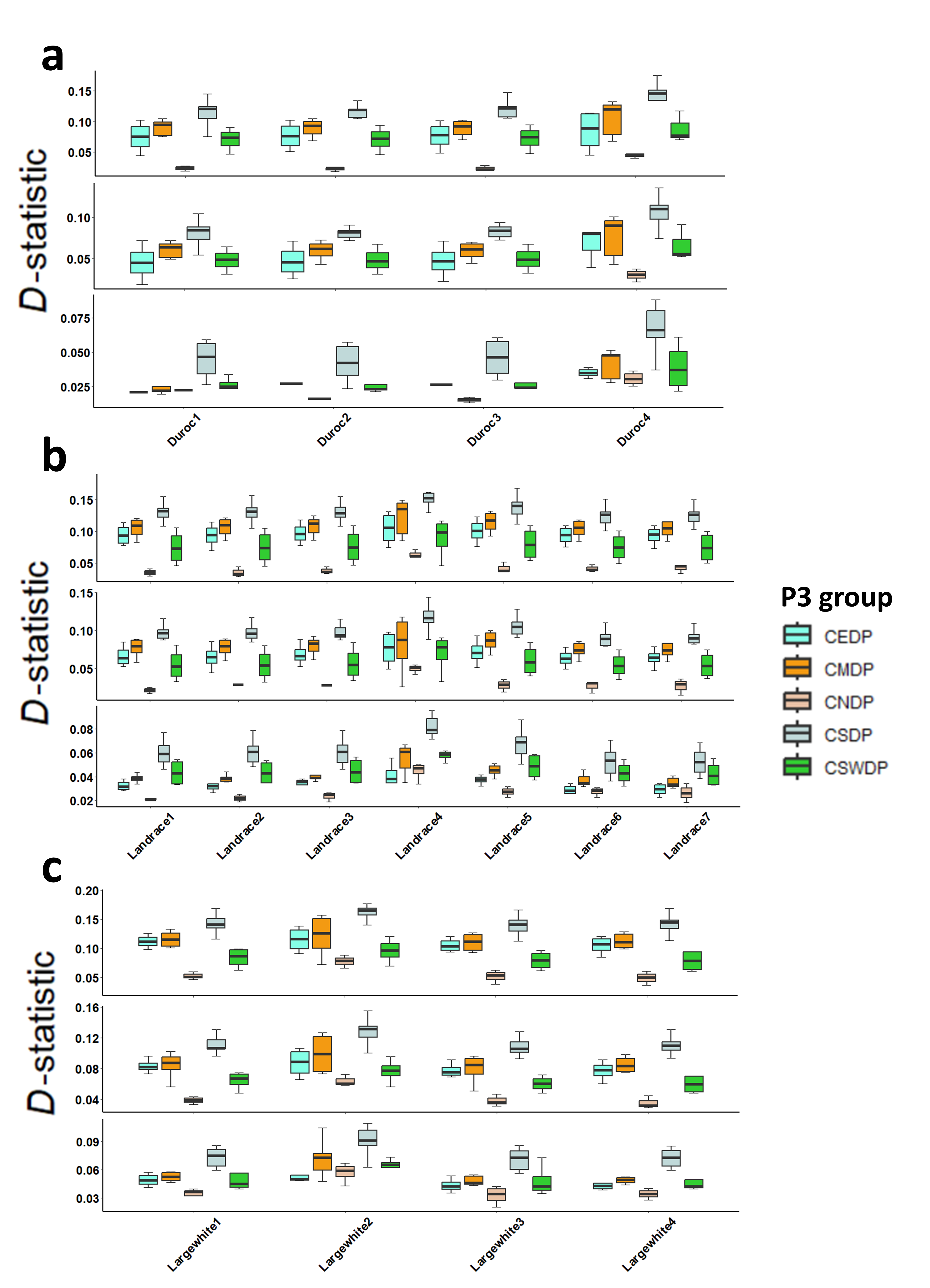


**Figure S1.** *D*-statistic summary of ECP in SNP chip data. (a) *D*-statistics of Duroc lines (Duroc), Duroc1: from Denmark; Duroc2: from the USA; Duroc3: from Netherlands; Duroc4: from China. (b) *D*-statistics of Landrace lines (Landrace), Landrace1: from Denmark; Landrace2: from Norway; Landrace3: from Finland; Landrace4: from China; Landrace5: from the USA; Landrace6: from Spain; Landrace7 from Netherlands. (c) *D*-statistics of Large White lines (Large White), Large White1: from Denmark; Large White2: from China; Large White3: from the USA; Large White4: from Netherlands.


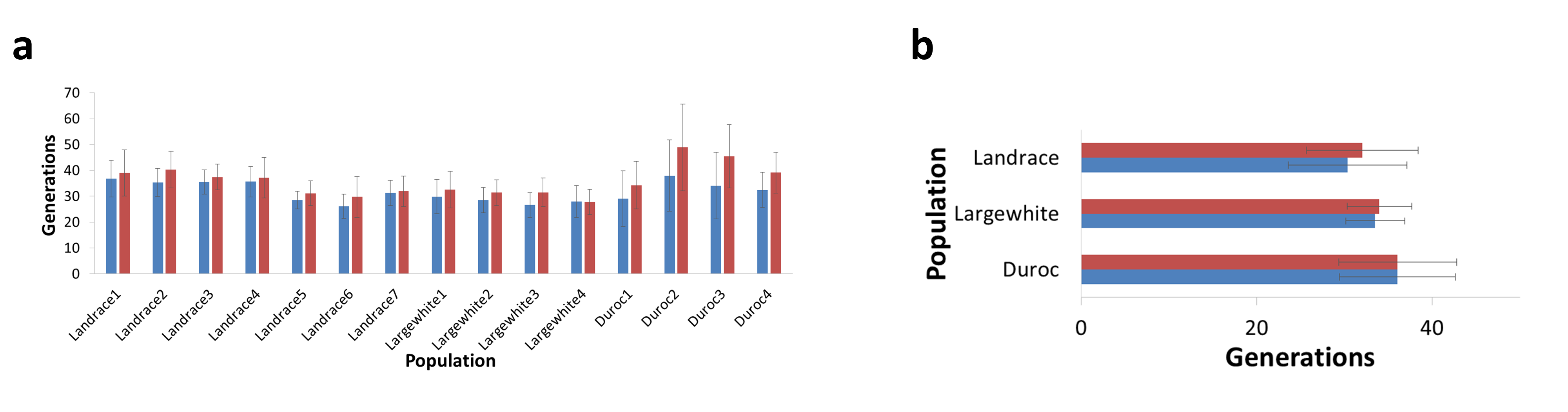


**Figure S2.** Estimated hybridization time between Chinese pigs (Eastern Chinese domestic pig: CEDP represents the blue color; Southern Chinese domestic pig: CSDP represents the red color) and ECP based on SNP chip data (a) and high-depth resequencing data (b).


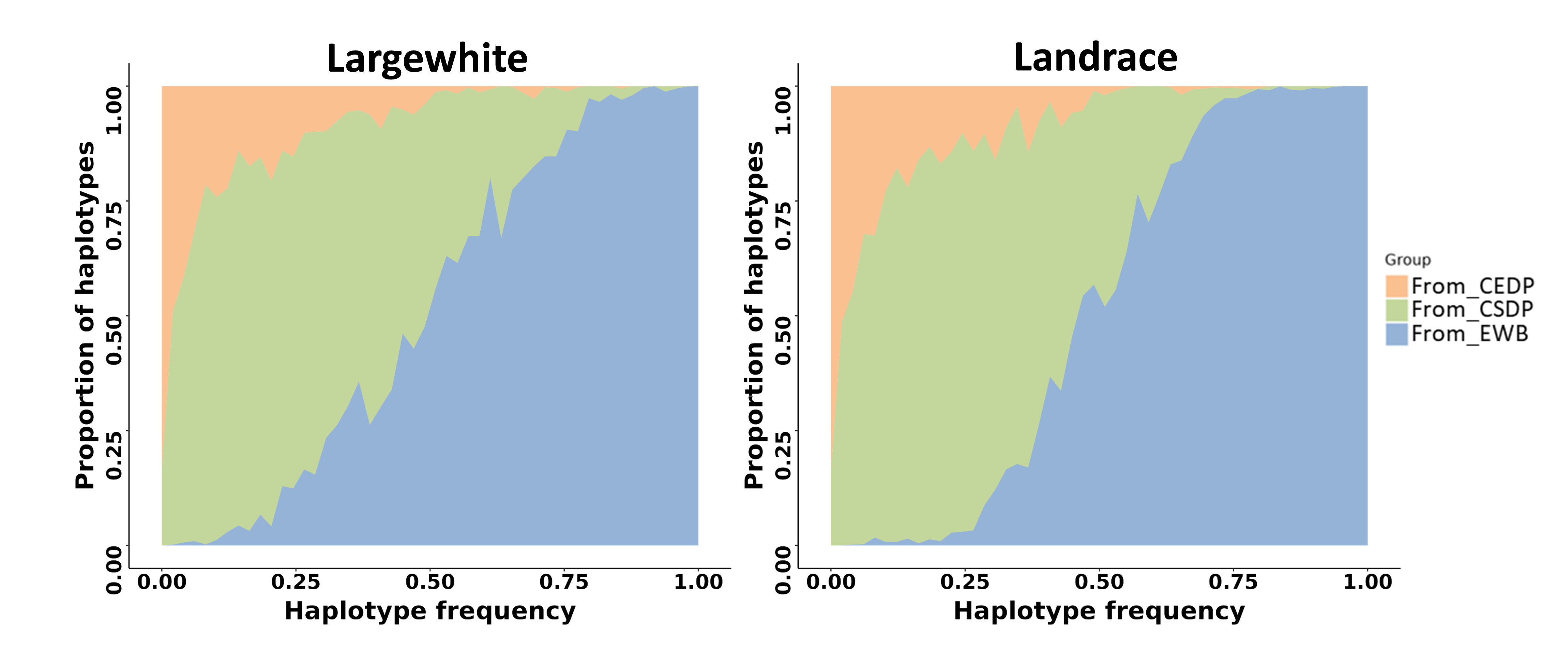


**Figure S3.** Diagram of local ancestry haplotype frequency in Large White and Landrace.


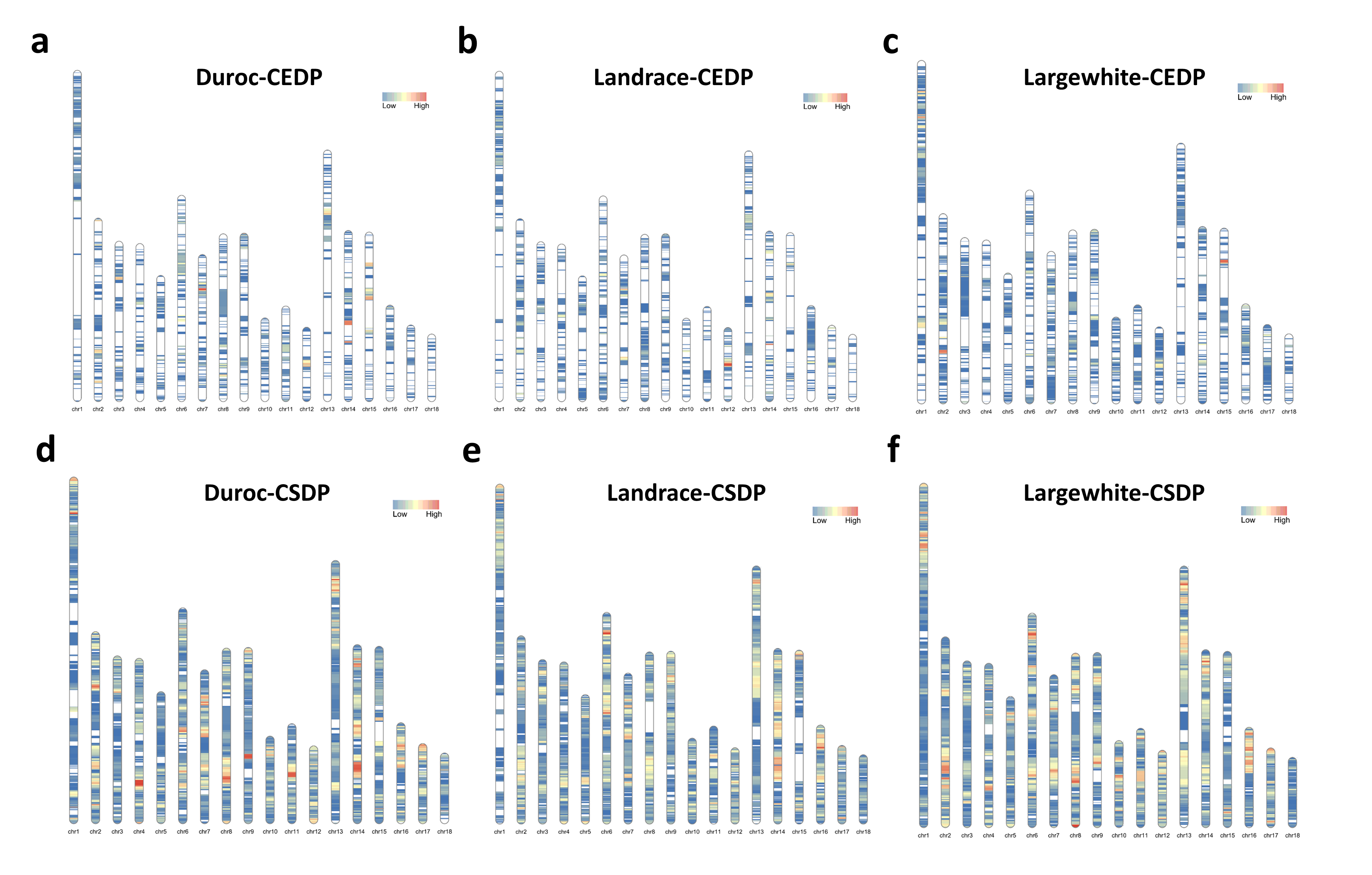


**Figure S4.** Genome-wide CEDP and CSDP-derived haplotype distribution in Duroc, Landrace and Large White.


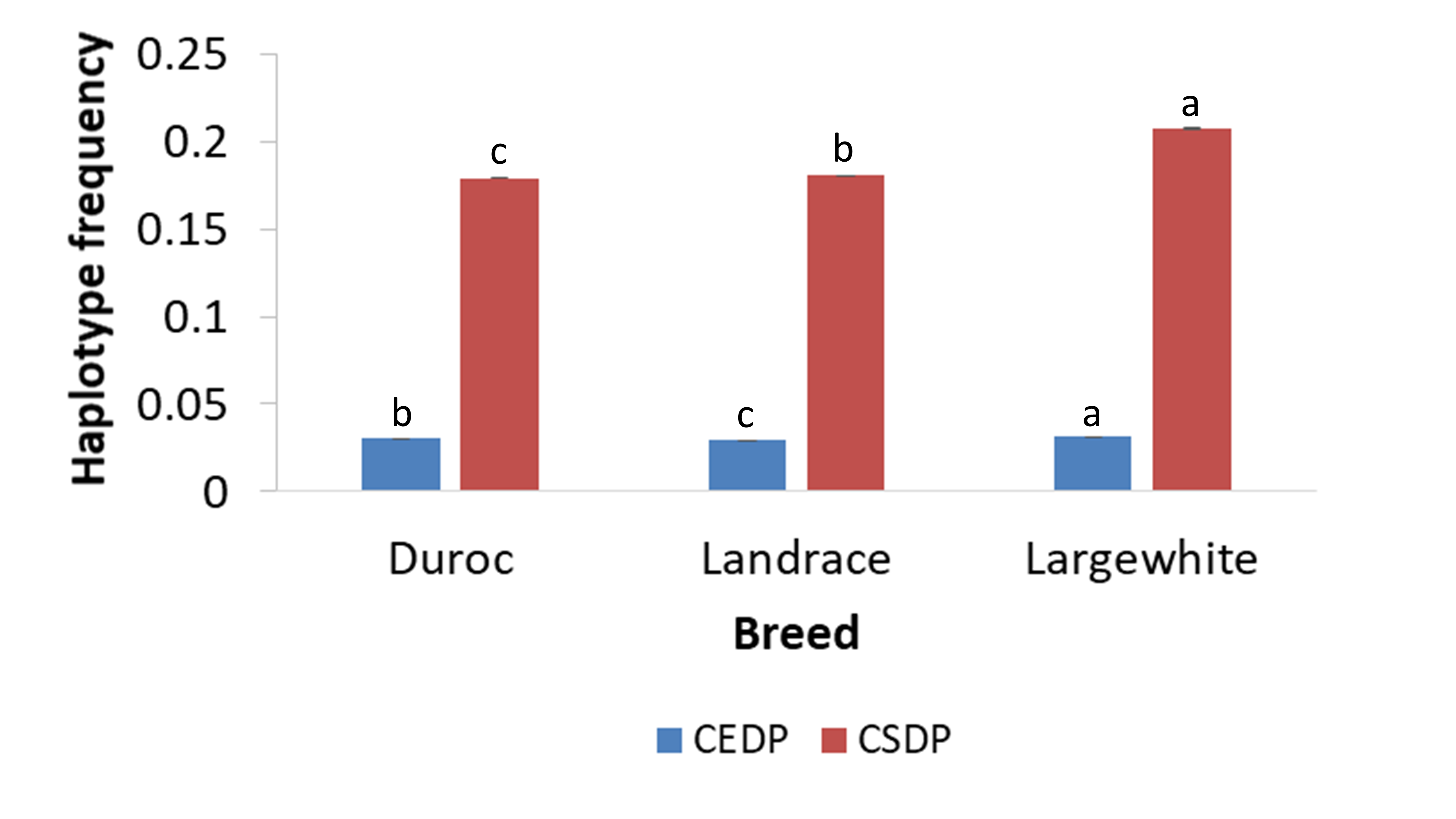


**Figure S5.** Average frequency of CEDP and CSDP-derived haplotype in Duroc, Landrace and Large White, and the 'a', 'b', and 'c' stand for the significance of variance test.


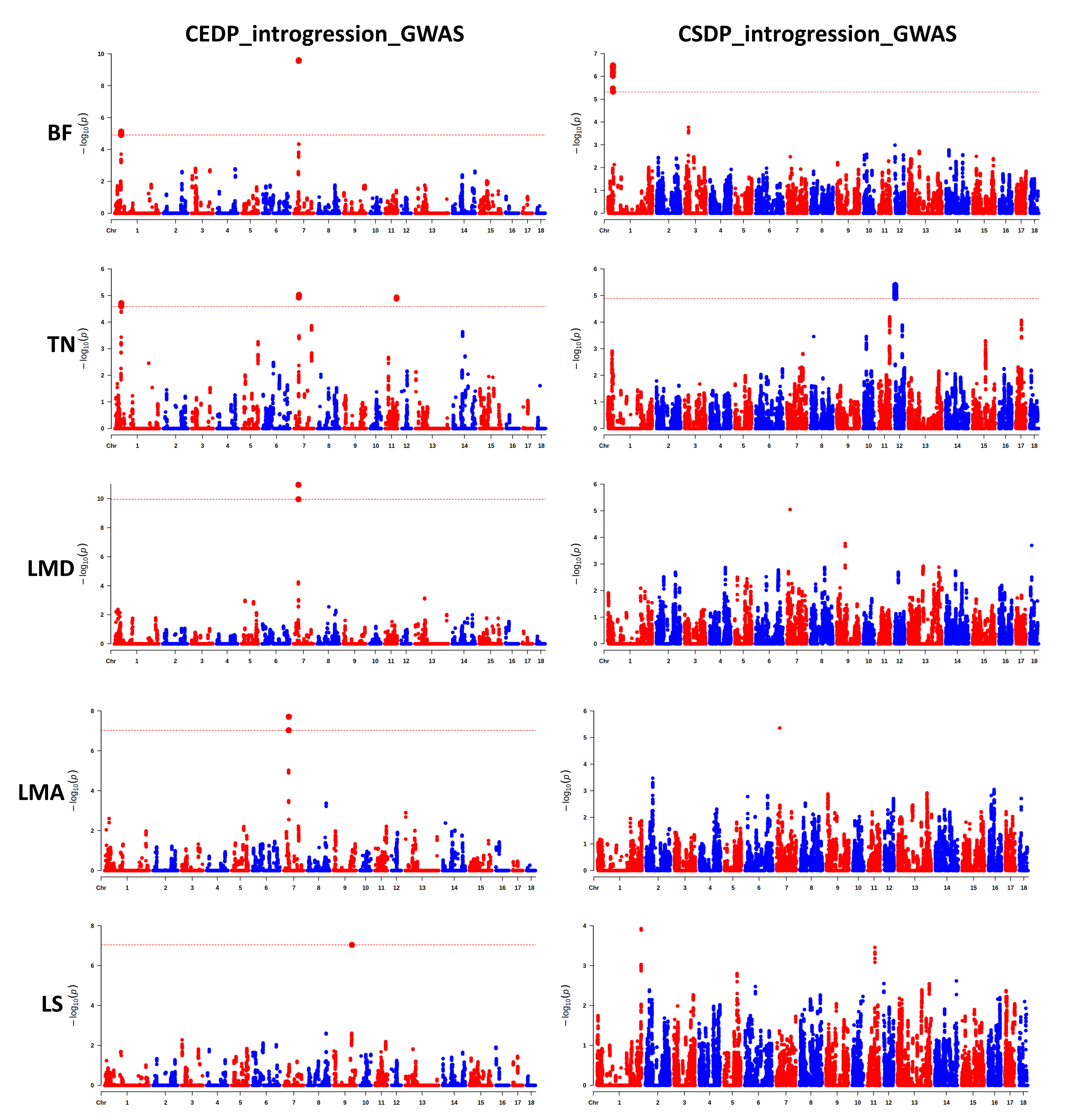


**Figure S6.** Manhattan plots for the individual traits from ancestral haplotype-based GWAS analysis.


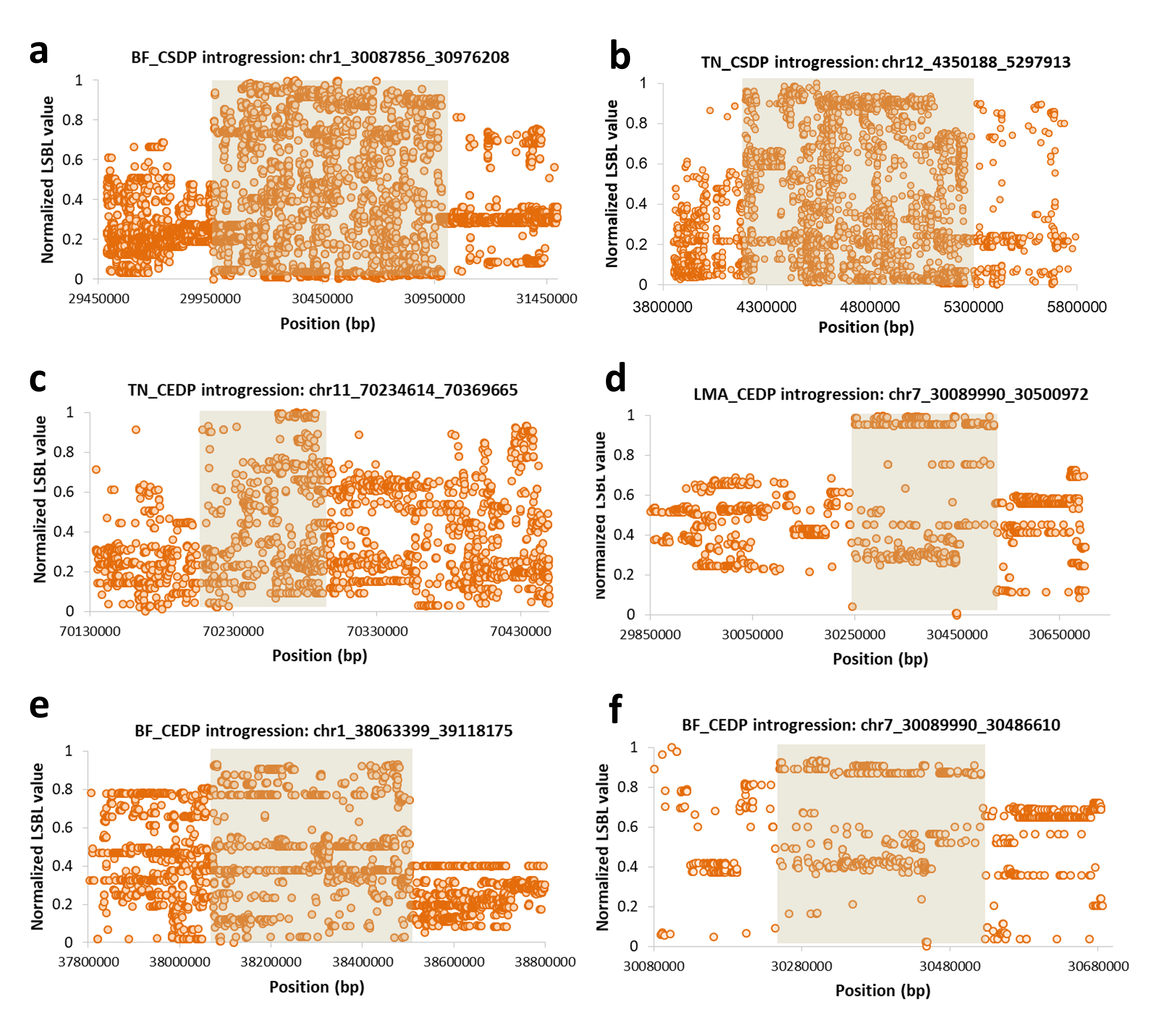


**Figure S7.** LSBL values of each SNP near the haplotype-based GWAS significant regions. The shaded region stands for the region of SNPs with highest LSBL values.


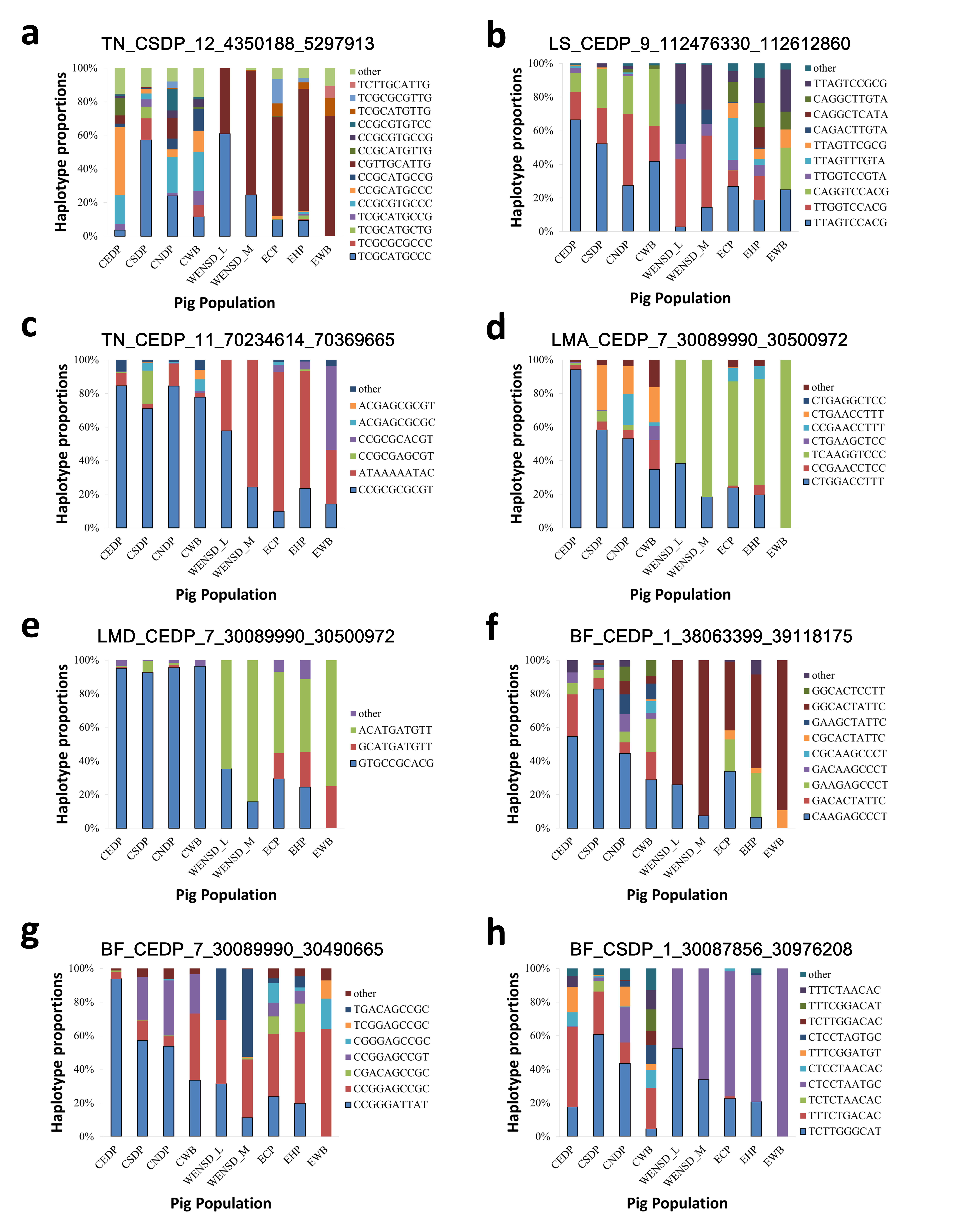


**Figure S8.** Haplotype frequencies in each populations for each QTL.


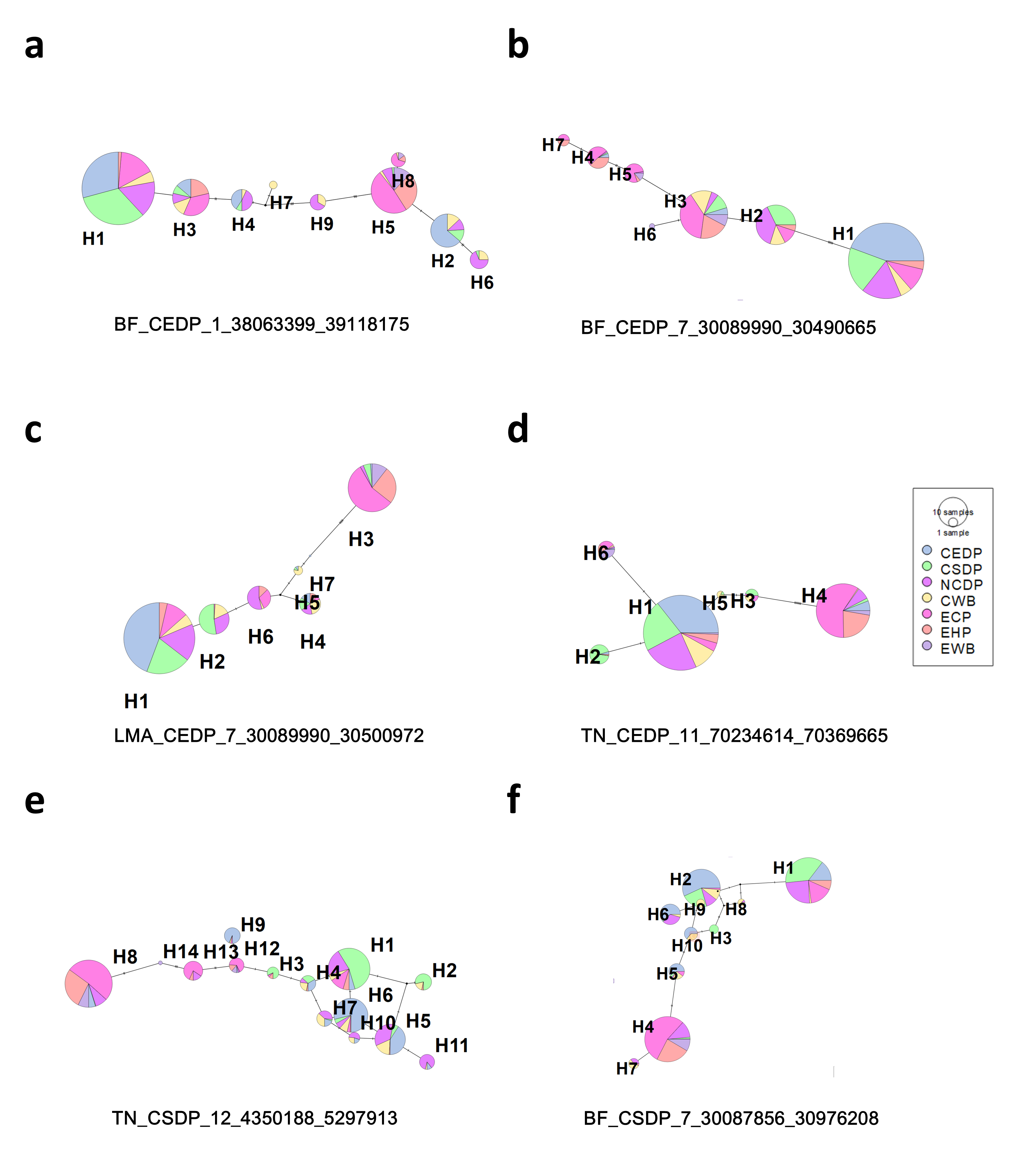


**Figure S9.** Haplotype network of the represented 10-SNP haplotype derived from each QTL.


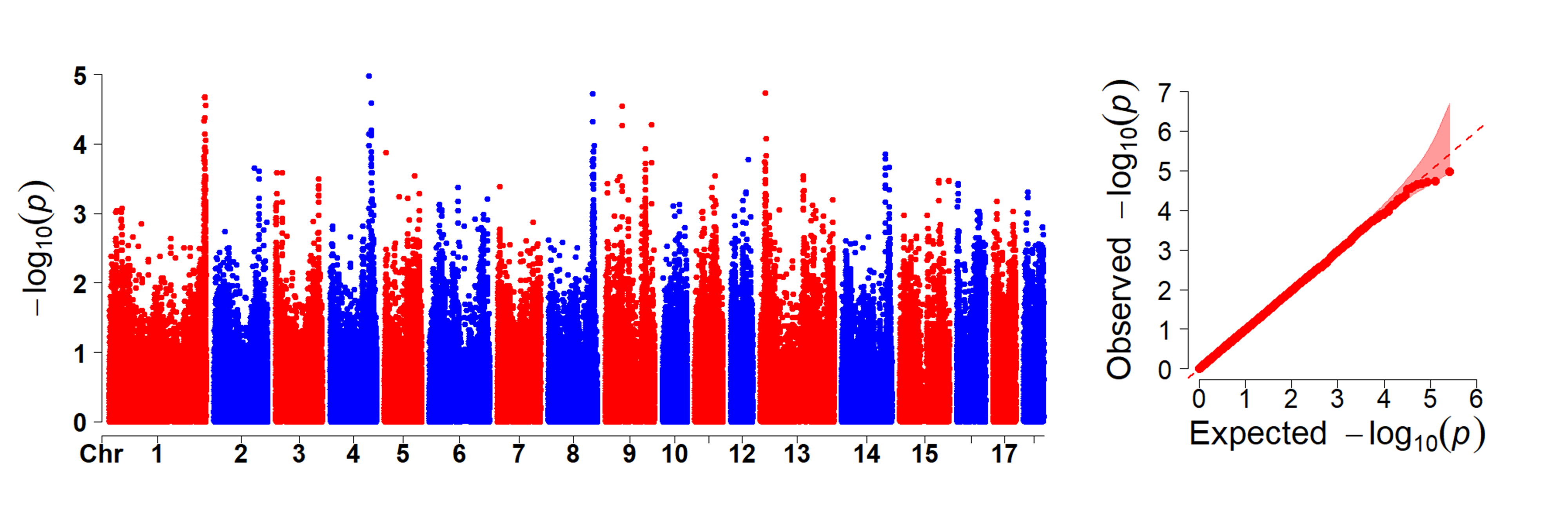


**Figure S10.** Manhattan and QQ plots of GWAS signals for litter size.


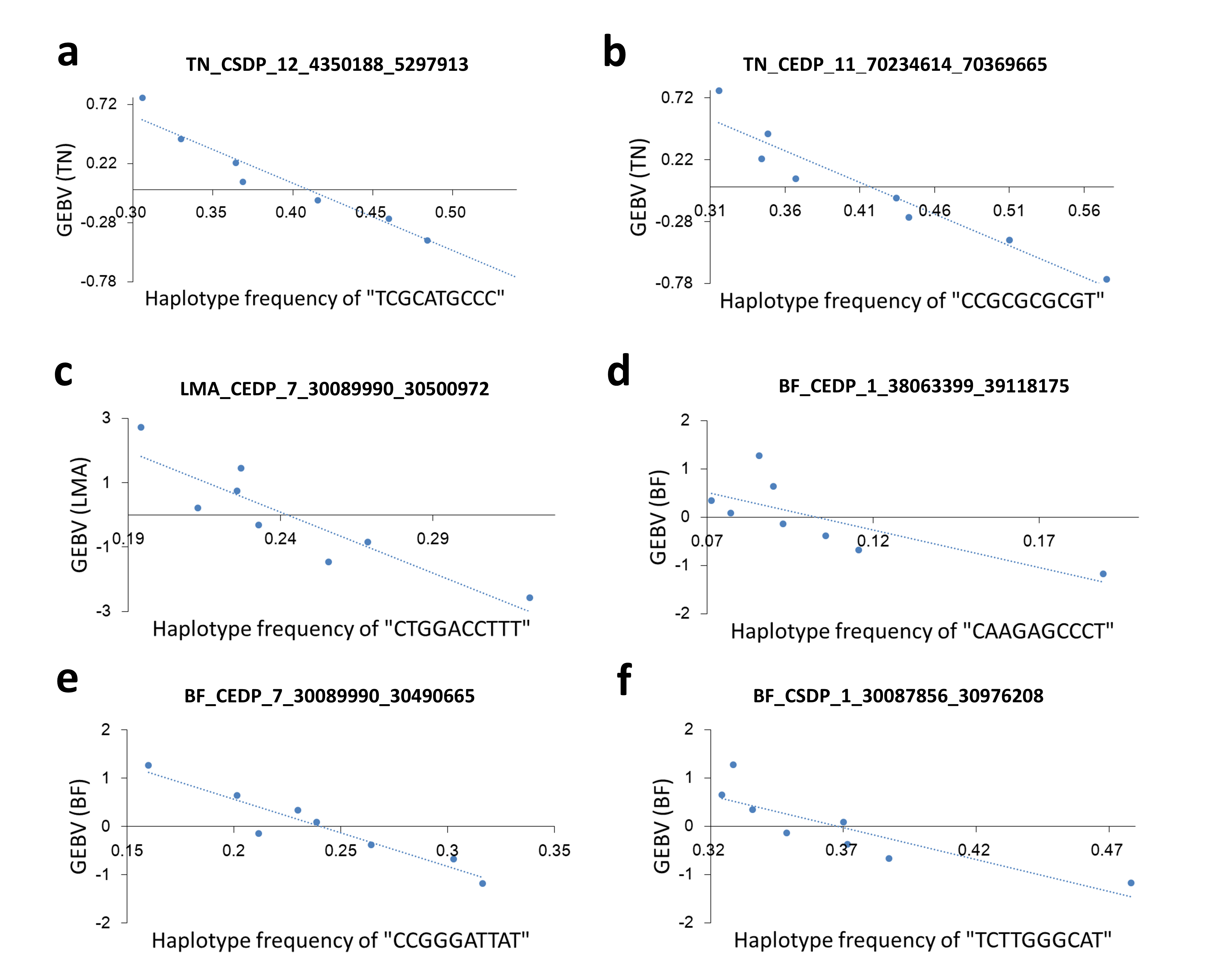


**Figure S11.** The relationship between the frequencies of haplotype derived from each QTL and GEBVs.


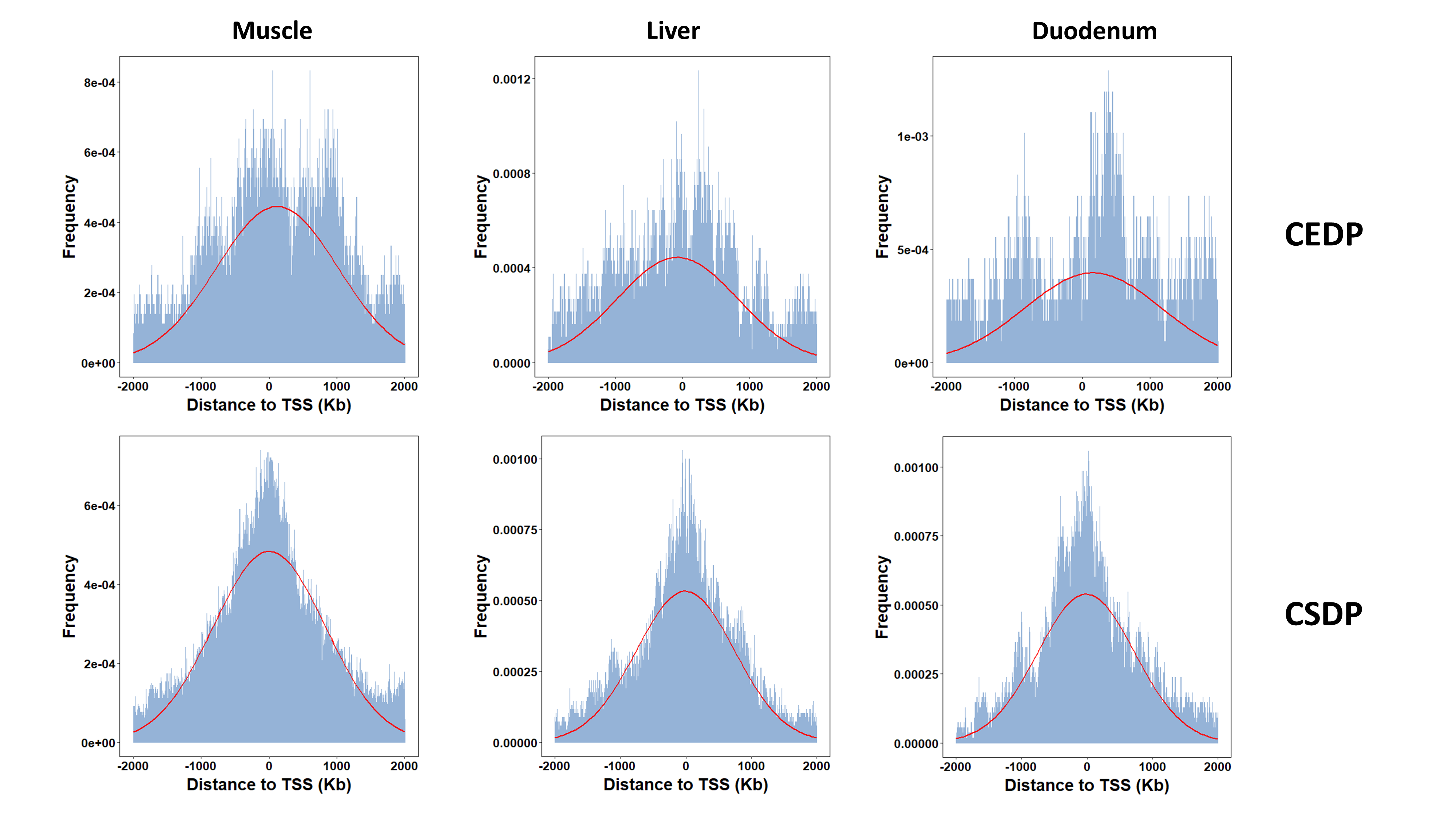


**Figure S12.** Density plot (red curve) of the distance of each significantly associated CEDP and CSDP-derived haplotype to the nearest transcript start site in muscle, liver, and duodenum tissues.


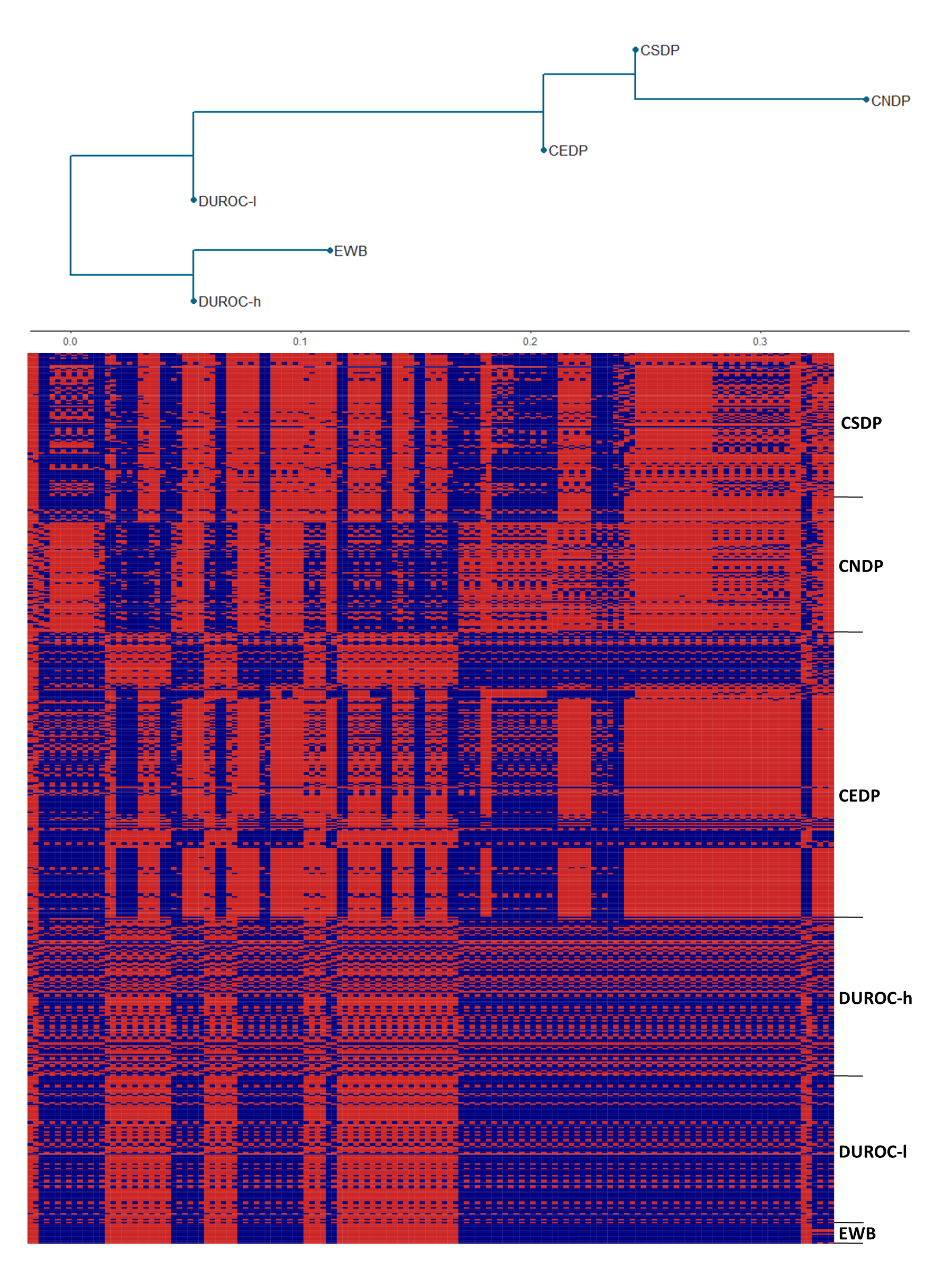


**Figure S13.** Maximum likelihood tree and heatmap of haplotype based on the 73 SNPs within the co-localized region.


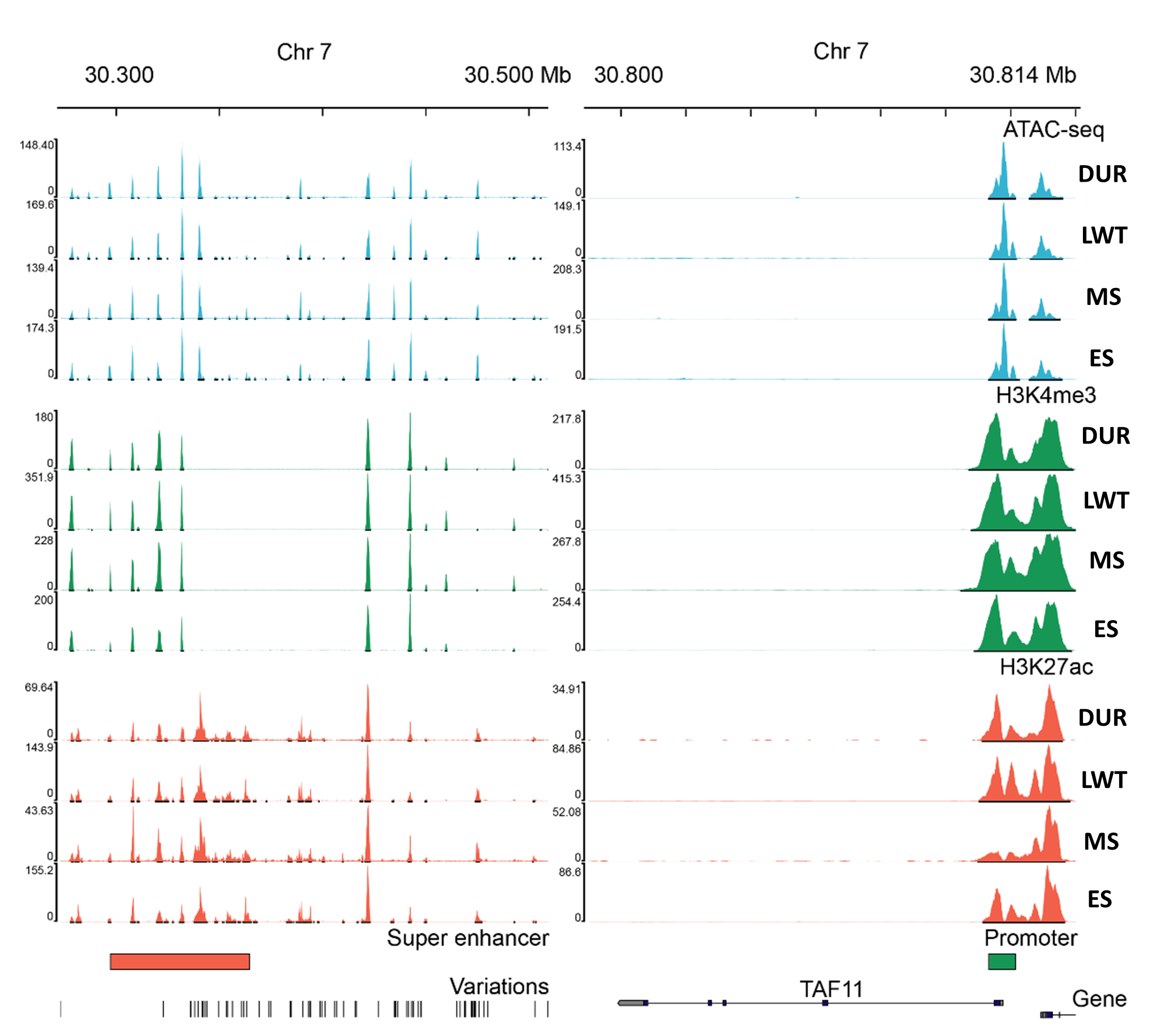


**Figure S14.** Peak calls of ATAC-seq and ChIP-seq using Duroc (DUR), Enshi (ES), Meishan (MS), and Large White (LWT) samples in the co-localized region and around *TAF11* region on Chromosome 7, where the red and green box represent the annotated super-enhancer, and promoter region of *TAF11*.


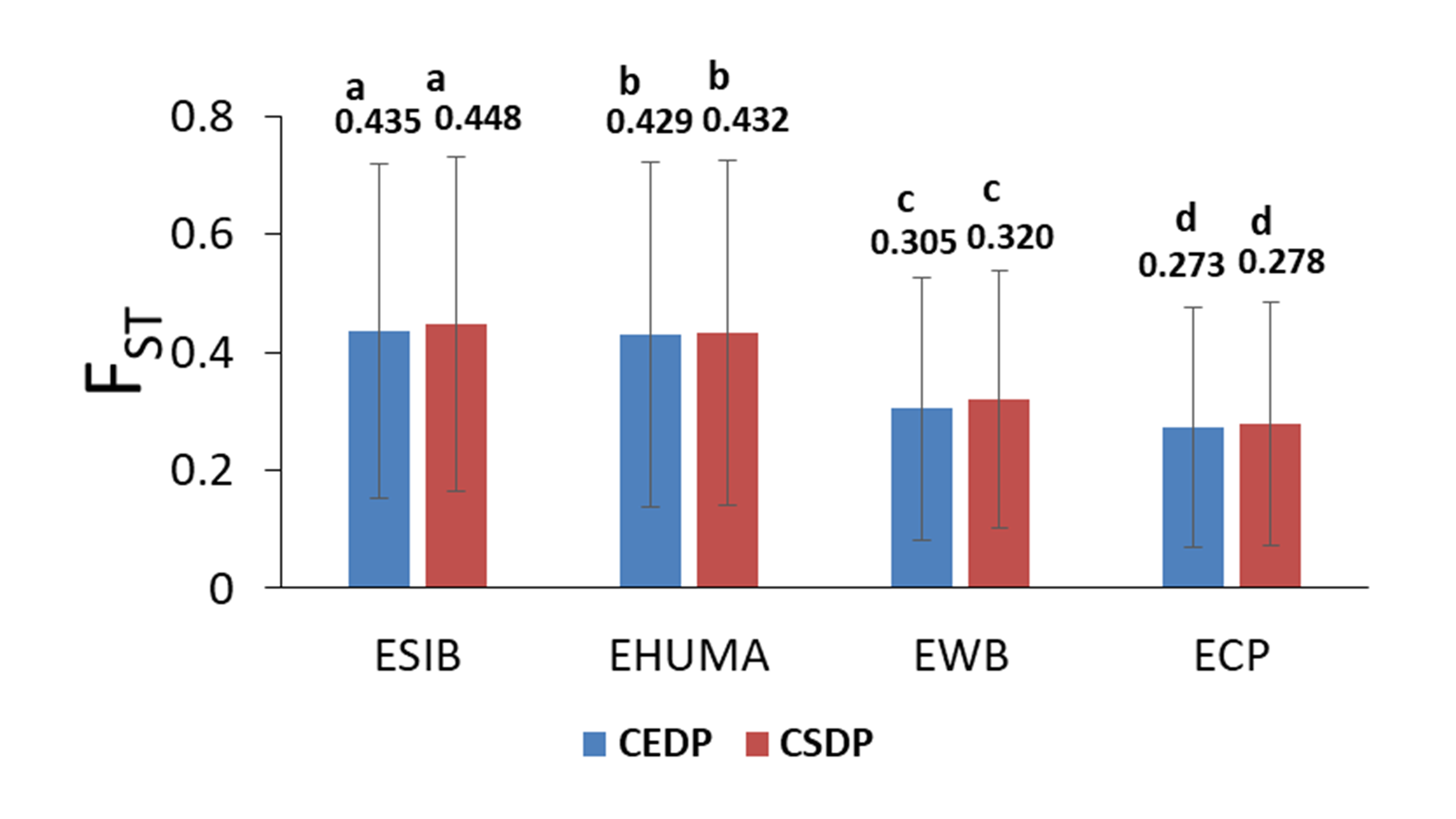


**Figure S15.** The comparisons of genetic differentiation between different European pig populations and Chinese pigs based on F_ST_. The 'a', 'b', 'c', and 'd' stand for the significance of variance test (Duncan-test). Error bars depict the standard deviations of F_ST_ statistic values.


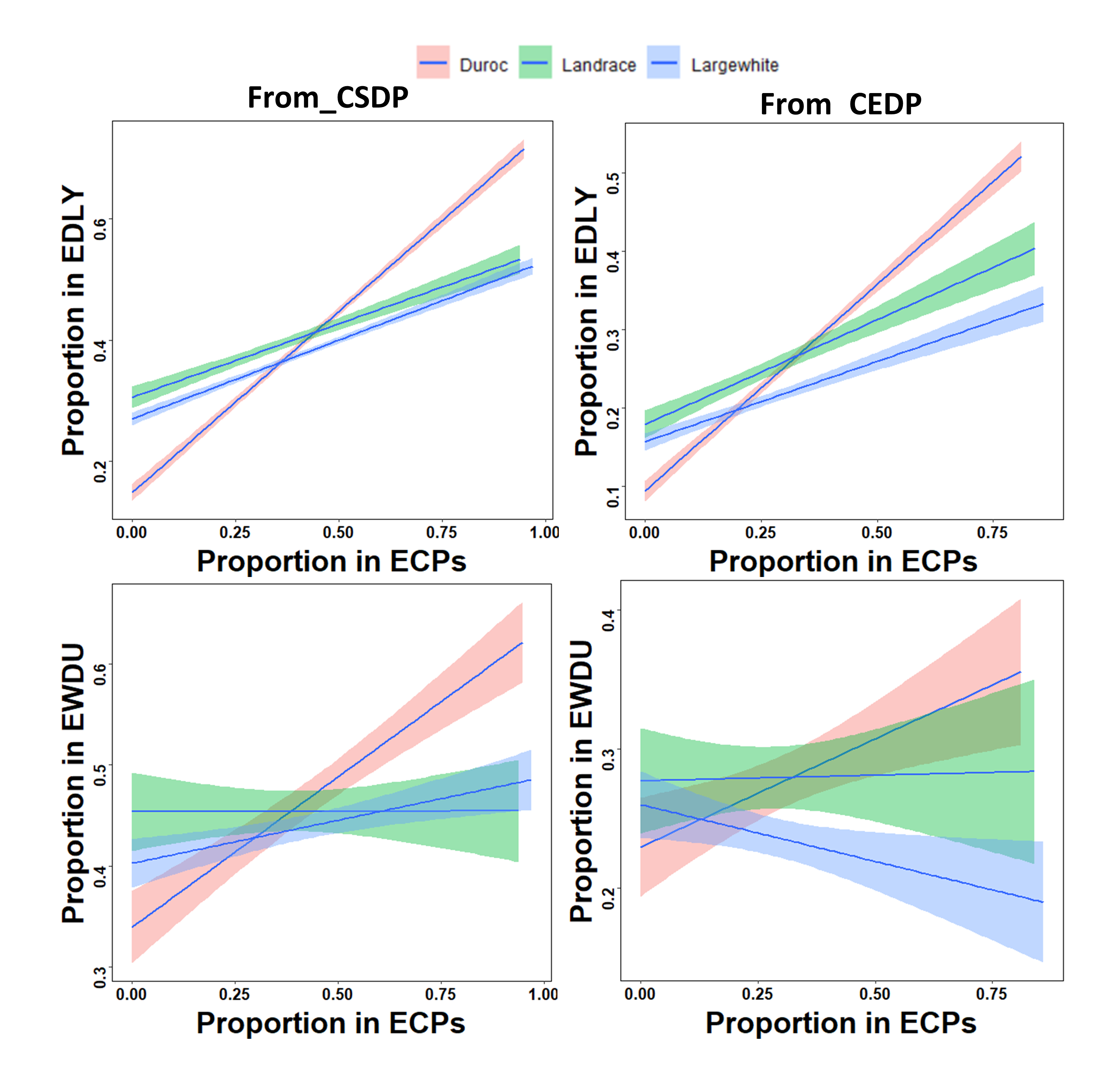


**Figure S16.** Linear fit between frequency of CEDP and CSDP-derived haplotype in ECP lines (Duroc, Landrace, and Largewhite) (x-axis) and EDLY (or EWDU) crossbred line (y-axis). The shaded area represents the 95% confidence interval around the fitted curve.


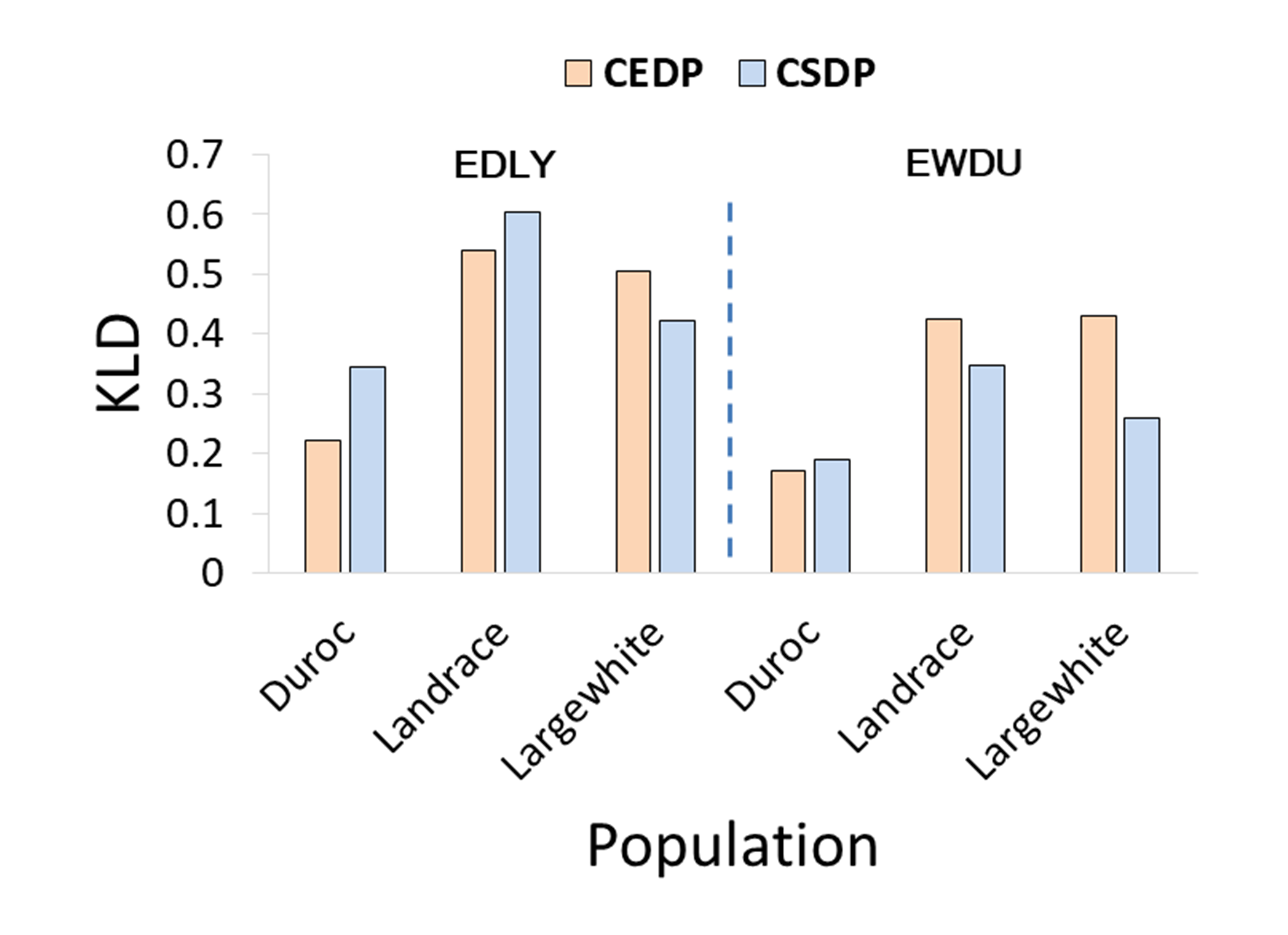


**Figure S17.** Kullback-Leibler divergence (KLD) results of CEDP and CSDP-derived haplotype frequency between ECP lines and crossbred lines (EDLY and EWDU).
